# Oxygen deprivation in breast cancer: mechanisms, pathways, and implications

**DOI:** 10.1097/MS9.0000000000003334

**Published:** 2025-04-25

**Authors:** Emmanuel Ifeanyi Obeagu

**Affiliations:** Department of Biomedical and Laboratory Science, Africa University, Mutare, Zimbabwe

**Keywords:** breast cancer, hypoxia, hypoxia-inducible factors, therapeutic resistance, tumor microenvironment

## Abstract

Hypoxia, a state of reduced oxygen availability, is a defining feature of the tumor microenvironment in breast cancer. It arises from the rapid proliferation of cancer cells, which outpaces the development of adequate vasculature. This oxygen deprivation triggers a cascade of molecular and cellular adaptations that enable tumor cells to survive and thrive under hostile conditions. Key among these is the stabilization of hypoxia-inducible factors, which regulate genes involved in angiogenesis, metabolic reprogramming, immune evasion, and cell survival. Hypoxia significantly influences breast cancer behavior, promoting tumor aggressiveness, therapeutic resistance, and metastatic potential. The hypoxic microenvironment fosters angiogenesis through vascular endothelial growth factor signaling, albeit leading to abnormal and inefficient vasculature. It also reprograms cancer cell metabolism towards glycolysis, supporting survival and growth in oxygen-deprived regions. Furthermore, hypoxia modulates immune responses, suppressing anti-tumor immunity while promoting the recruitment of immunosuppressive cells. These multifaceted effects underscore hypoxia’s pivotal role in shaping the clinical trajectory of breast cancer.

## Introduction

Breast cancer remains a leading cause of cancer-related morbidity and mortality worldwide, with its incidence steadily rising despite advances in early detection and treatment. Among the various factors influencing breast cancer progression, the tumor microenvironment plays a pivotal role in dictating its aggressiveness and therapy response. Hypoxia, defined as a reduction in oxygen availability below physiological levels, is a hallmark of the tumor microenvironment and a critical determinant of breast cancer behavior. It arises due to the imbalance between oxygen demand and supply, resulting from rapid tumor cell proliferation and insufficient vascularization^[1]^. The hypoxic tumor microenvironment is not merely a byproduct of tumor growth but an active driver of malignant progression. Under hypoxic conditions, cancer cells undergo extensive molecular and cellular adaptations to survive and thrive. Central to this response is the stabilization and activation of HIFs, a family of transcription factors that regulate the expression of genes involved in angiogenesis, metabolism, immune evasion, and metastasis. These adaptive mechanisms enable breast cancer cells to overcome the challenges posed by oxygen deprivation while creating an environment conducive to tumor progression^[2]^. Hypoxia exerts profound effects on tumor biology, influencing processes such as angiogenesis, metabolic reprogramming, immune modulation, and therapy resistance. Angiogenesis, driven predominantly by vascular endothelial growth factor (VEGF) signaling, represents the tumor’s attempt to restore oxygen supply. However, the resulting vasculature is often abnormal and inefficient, perpetuating a cycle of hypoxia. Metabolically, hypoxic breast cancer cells shift toward glycolysis for energy production, even in the presence of oxygen, a phenomenon known as the Warburg effect. This metabolic reprogramming not only supports survival but also creates a more acidic and invasive tumor microenvironment^[3]^.

**HIGHLIGHTS**

- Oxygen deprivation (hypoxia) accelerates tumor progression and metastasis in breast cancer.
- HIFs activate survival, angiogenesis, and metabolic pathways under low oxygen conditions.
- Hypoxia enhances chemotherapy resistance and immune evasion in tumors.
- Altered metabolism under hypoxia supports cancer cell survival.
- Targeting hypoxia-related pathways offers potential for novel therapeutic strategies.

The immune landscape of breast cancer is also profoundly altered by hypoxia. Oxygen deprivation suppresses the activity of cytotoxic T cells and natural killer (NK) cells, while promoting the recruitment and activation of immunosuppressive cells such as regulatory T cells and myeloid-derived suppressor cells (MDSCs). This immunosuppressive microenvironment enables cancer cells to evade immune surveillance, further enhancing their survival and metastatic potential. Additionally, hypoxia has been shown to drive epithelial-to-mesenchymal transition (EMT), a process critical for invasion and metastasis, by modulating pathways such as Notch, Wnt, and TGF-β^[4,5]^. Therapeutic resistance is another major consequence of hypoxia in breast cancer. Hypoxic regions within tumors are less sensitive to radiotherapy due to reduced oxygen availability, which limits the generation of reactive oxygen species needed for effective DNA damage. Similarly, chemotherapy efficacy is compromised in hypoxic environments, as these regions often harbor dormant cancer cells that are less responsive to cytotoxic agents. Together, these factors contribute to the poor prognosis associated with hypoxic tumors, highlighting the need for effective strategies to target hypoxia^[6,7]^. Despite the challenges posed by hypoxia, it also presents opportunities for therapeutic intervention. Hypoxia-activated prodrugs, designed to be selectively activated in oxygen-deprived conditions, have shown promise in targeting hypoxic tumor regions. Similarly, anti-angiogenic therapies aim to normalize tumor vasculature and reduce hypoxia, though their effectiveness in breast cancer remains a subject of debate. The inhibition of HIFs and other hypoxia-associated pathways offers another avenue for disrupting the adaptive mechanisms employed by hypoxic cancer cells^[8]^. Emerging strategies also include the integration of immunotherapies with hypoxia-targeting agents. By alleviating hypoxia-induced immunosuppression, these approaches seek to restore the anti-tumor immune response while simultaneously targeting cancer cell survival pathways. Additionally, biomarkers of hypoxia, such as HIF expression levels and hypoxia gene signatures, are being explored for their potential to guide patient stratification and treatment planning^[9]^.

### Aim

The aim of this review article is to provide a comprehensive overview of the mechanisms and implications of oxygen deprivation (hypoxia) in breast cancer, explore the pathways activated by hypoxic conditions, and discuss the therapeutic strategies targeting hypoxia for improving treatment outcomes.

### Justification of the review

Breast cancer remains one of the leading causes of cancer-related deaths worldwide, despite significant advances in early detection and treatment. One of the key factors contributing to poor prognosis and resistance to therapy in breast cancer is the tumor’s ability to adapt to low oxygen conditions, known as hypoxia. Hypoxia plays a critical role in tumor progression by driving metabolic reprogramming, angiogenesis, immune evasion, and increased metastasis, all of which contribute to therapy resistance and unfavorable outcomes. Despite the growing body of research on hypoxia in cancer, the clinical translation of hypoxia-targeting therapies has been slow, with many strategies still in experimental stages. Current therapeutic options are limited in their ability to address the complexities of hypoxia within the tumor microenvironment. By reviewing the existing literature on the molecular mechanisms, pathways activated by hypoxia, and potential therapeutic interventions, this review aims to provide an in-depth understanding of the role of hypoxia in breast cancer and to highlight the most promising approaches for future clinical applications.^[1-4]^ Additionally, the review will contribute to filling knowledge gaps in hypoxia-driven breast cancer therapy, potentially guiding the development of new treatment paradigms and improving patient outcomes.

## Review methods

### Literature search strategy

A detailed literature search was conducted using major electronic databases, including PubMed, Scopus, Google Scholar, and Web of Science, to identify relevant articles. The search terms included “hypoxia in breast cancer,” “oxygen deprivation,” “tumor microenvironment,” “hypoxia-induced pathways,” “hypoxia-targeted therapies in breast cancer,” and “hypoxia-driven metastasis.”

The search was limited to peer-reviewed journal articles, including original research studies, reviews, and clinical trial reports. Additionally, key references from articles were manually reviewed for inclusion to ensure that all relevant studies were considered.

### Inclusion and exclusion criteria

The following inclusion criteria were used to select articles for review:

- Studies focusing on the role of hypoxia in breast cancer biology, including molecular mechanisms, pathways, and clinical implications.
- Research on therapeutic strategies targeting hypoxia in breast cancer treatment.
- Review articles and meta-analyses that provide comprehensive summaries of hypoxia-related breast cancer mechanisms and treatments.

Exclusion criteria:

- Studies unrelated to breast cancer or focusing on other cancer types.
- Studies with inadequate methodological rigor or lacking detailed results.
- Articles that do not specifically address the role of hypoxia in tumor progression or treatment.

### Mechanisms of hypoxia in breast cancer

Hypoxia in breast cancer is a result of rapid tumor growth outpacing the development of an adequate blood supply. This imbalance creates a microenvironment where oxygen levels drop significantly, triggering a cascade of molecular and cellular adaptations. These adaptations enable tumor cells to survive, proliferate, and evade therapy under oxygen-deprived conditions. The primary mechanisms through which hypoxia influences breast cancer include hypoxia-inducible factors (HIFs), angiogenesis, metabolic reprogramming, immune modulation, and EMT.

1. **Hypoxia-Inducible Factors (HIFs)**

HIFs are the central mediators of cellular responses to hypoxia. Under normoxic conditions, HIF-1α and HIF-2α are degraded by the ubiquitin-proteasome pathway. However, in hypoxic conditions, these factors are stabilized and translocate to the nucleus, where they dimerize with HIF-1β and activate the transcription of genes involved in survival, angiogenesis, and metabolism. HIF-1α, in particular, promotes the expression of VEGF, glucose transporter 1 (GLUT1), and enzymes associated with glycolysis. In breast cancer, elevated HIF levels are associated with increased tumor invasiveness, metastasis, and resistance to therapy^[5]^.

**2. Angiogenesis**

Oxygen deprivation induces angiogenesis, the formation of new blood vessels, as a compensatory mechanism to restore oxygen delivery. Hypoxia-driven stabilization of HIF-1α leads to the upregulation of VEGF, a potent pro-angiogenic factor. VEGF stimulates the proliferation and migration of endothelial cells, resulting in the formation of new vasculature. However, tumor-induced angiogenesis often produces aberrant and dysfunctional blood vessels that are leaky and poorly organized. This perpetuates hypoxia, creating a feedback loop that further exacerbates tumor progression^[10]^.

**3. Metabolic Reprogramming**

Hypoxia triggers a metabolic shift in breast cancer cells from oxidative phosphorylation to glycolysis, a phenomenon known as the Warburg effect. This adaptation, driven by HIFs, enables cancer cells to generate energy and biosynthetic precursors under low oxygen conditions. Key enzymes such as lactate dehydrogenase A (LDHA) are upregulated, leading to the accumulation of lactate, which acidifies the tumor microenvironment. This acidic environment promotes immune evasion, matrix remodeling, and invasion, thereby enhancing tumor aggressiveness^[11]^.

**4. Immune Modulation**

The hypoxic tumor microenvironment exerts profound immunosuppressive effects. Hypoxia impairs the function of cytotoxic T cells and NK cells, reducing their ability to eliminate tumor cells. Simultaneously, it recruits immunosuppressive cells such as regulatory T cells (Tregs) and MDSCs through chemokines like CCL28. Hypoxia also promotes the expression of immune checkpoint molecules such as programmed death-ligand 1 (PD-L1), further dampening anti-tumor immunity. These mechanisms create an immune-privileged niche that supports tumor survival and progression^[12]^.

**5. Epithelial-to-Mesenchymal Transition (EMT)**

Hypoxia plays a critical role in driving EMT, a process through which epithelial cancer cells acquire mesenchymal characteristics, enhancing their motility and invasiveness. HIF-1α modulates EMT by upregulating transcription factors such as Snail, Twist, and Zeb1, which suppress E-cadherin expression and promote the expression of mesenchymal markers like N-cadherin and vimentin. EMT facilitates tumor cell detachment from the primary tumor, invasion into surrounding tissues, and eventual metastasis to distant organs^[13]^.

**6. DNA Damage and Genomic Instability**

Hypoxia contributes to genomic instability by impairing DNA damage repair pathways. Under oxygen-deprived conditions, the activity of DNA repair enzymes such as RAD51 is reduced, leading to the accumulation of DNA damage. This genomic instability drives tumor heterogeneity, promoting the selection of aggressive and therapy-resistant clones^[13]^.

**7. Hypoxia-Induced Stemness**

Hypoxia has been shown to enhance the stemness of breast cancer cells, contributing to their ability to self-renew and initiate tumors. HIFs upregulate stemness-associated genes, such as OCT4, SOX2, and NANOG, and promote the enrichment of cancer stem cell populations within hypoxic regions of tumors. These stem-like cells are highly resistant to conventional therapies and are thought to play a pivotal role in tumor recurrence^[8]^.

**8. Resistance to Therapy**

**Figure 1.**
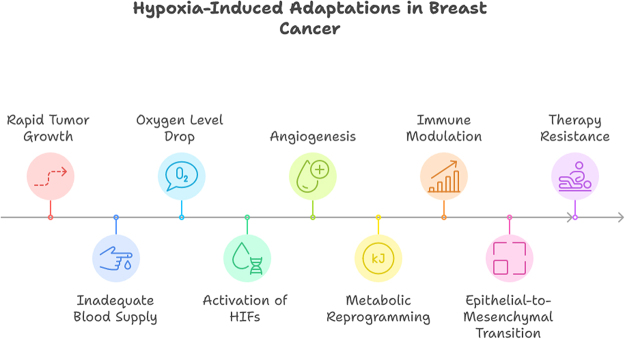
Hypoxia-induced adaptations in breast cancer.

Hypoxic breast cancer cells exhibit resistance to radiotherapy and chemotherapy. The lack of oxygen reduces the efficacy of radiation therapy, which relies on the generation of reactive oxygen species to induce DNA damage. Hypoxia also creates a quiescent cancer cell population that is less sensitive to chemotherapeutic agents targeting actively dividing cells^[8]^ (Figure 1).

### Pathways activated by hypoxia in breast cancer

Hypoxia in breast cancer activates several signaling pathways that regulate cellular and molecular processes critical for tumor survival, progression, and therapy resistance. These pathways are primarily mediated by HIFs and their downstream targets.

1. **Hypoxia-Inducible Factor (HIF) Signaling Pathway**

The HIF pathway is the central mediator of cellular responses to hypoxia. Under normoxic conditions, HIF-α subunits (HIF-1α and HIF-2α) are hydroxylated by prolyl hydroxylases (PHDs), marking them for ubiquitination and proteasomal degradation. In hypoxia, PHDs are inactivated due to low oxygen availability, allowing HIF-α stabilization and nuclear translocation. HIF-α binds with HIF-1β and co-activators such as p300/CBP to form a transcriptional complex that activates hypoxia-responsive elements (HREs) in the promoters of target genes. These genes regulate processes like angiogenesis (VEGF), metabolism (GLUT1, LDHA), and survival (BNIP3), which are critical for breast cancer progression^[14,15]^.

**2. Vascular Endothelial Growth Factor (VEGF) Pathway**

Hypoxia induces VEGF expression through HIF-1α activation. VEGF is a potent pro-angiogenic factor that promotes endothelial cell proliferation, migration, and new blood vessel formation. The VEGF pathway signals through VEGF receptors (VEGFRs) on endothelial cells, activating downstream pathways such as PI3K/AKT and MAPK. While angiogenesis is essential for delivering oxygen and nutrients, the abnormal vasculature it generates often exacerbates hypoxia, perpetuating tumor progression and creating a feedback loop^[16]^.

**3. PI3K/AKT/mTOR Pathway**

The phosphatidylinositol-3-kinase (PI3K)/AKT/mammalian target of rapamycin (mTOR) pathway is frequently activated in hypoxic breast cancer cells. HIF-1α induces the expression of growth factors like insulin-like growth factor 1 (IGF-1) and VEGF, which activate PI3K signaling. AKT phosphorylation promotes cell survival by inhibiting pro-apoptotic proteins such as BAD and caspase-9. Additionally, mTOR signaling enhances protein synthesis and cell growth, enabling tumor adaptation to hypoxic stress^[17]^.

**4. MAPK/ERK Pathway**

The mitogen-activated protein kinase (MAPK)/extracellular signal-regulated kinase (ERK) pathway is activated in response to hypoxia and contributes to tumor cell proliferation and invasion. Hypoxia-induced HIF-1α expression upregulates growth factors like EGF and TGF-α, which bind to their respective receptors and activate the MAPK/ERK pathway. This signaling cascade enhances the expression of matrix metalloproteinases (MMPs), facilitating extracellular matrix degradation and metastasis^[18]^.

**5. Notch Pathway**

The Notch signaling pathway is another key mediator of hypoxia-driven tumor progression. Hypoxia activates Notch signaling by inducing the expression of Notch ligands such as Delta-like 4 (DLL4) through HIF-1α. The interaction between Notch ligands and receptors leads to the cleavage of the Notch intracellular domain (NICD), which translocates to the nucleus and regulates genes involved in stemness, EMT, and angiogenesis. Notch signaling plays a pivotal role in maintaining cancer stem cell populations and promoting resistance to therapy^[19]^.

**6. NF-κB Pathway**

The nuclear factor kappa-light-chain-enhancer of activated B cells (NF-κB) pathway is activated under hypoxic conditions and contributes to inflammation, survival, and immune evasion in breast cancer. HIF-1α interacts with NF-κB to enhance the expression of pro-survival and pro-inflammatory genes, including IL-6 and TNF-α. These cytokines recruit immunosuppressive cells and create a tumor-promoting microenvironment^[20]^.

**7. Wnt/β-Catenin Pathway**

Hypoxia activates the Wnt/β-catenin signaling pathway, which regulates cell proliferation, migration, and stemness. HIF-1α stabilizes β-catenin by inhibiting glycogen synthase kinase-3β (GSK-3β)-mediated degradation. Stabilized β-catenin translocates to the nucleus and activates transcription of genes associated with EMT, invasion, and metastasis, such as c-MYC and cyclin D1^[21]^.

**8. TGF-β Pathway**

The transforming growth factor-beta (TGF-β) pathway is modulated by hypoxia and contributes to EMT and metastasis. Hypoxia increases TGF-β expression, which activates Smad-dependent and Smad-independent pathways. These pathways downregulate E-cadherin, a key epithelial marker, while upregulating mesenchymal markers like vimentin, enhancing the migratory and invasive capabilities of cancer cells^[22]^.

**9. Apoptosis and Autophagy Pathways**

Hypoxia induces both pro-survival and pro-death mechanisms in breast cancer. HIF-1α promotes the expression of pro-survival factors like BCL-2 and BNIP3, enabling cancer cells to evade apoptosis. Simultaneously, hypoxia triggers autophagy as a stress response, allowing cells to recycle damaged organelles and sustain energy production. While autophagy is initially protective, excessive activation can lead to cell death, highlighting its dual role in hypoxic tumors^[23]^.

**10. Epigenetic Regulation**

**Figure 2.**
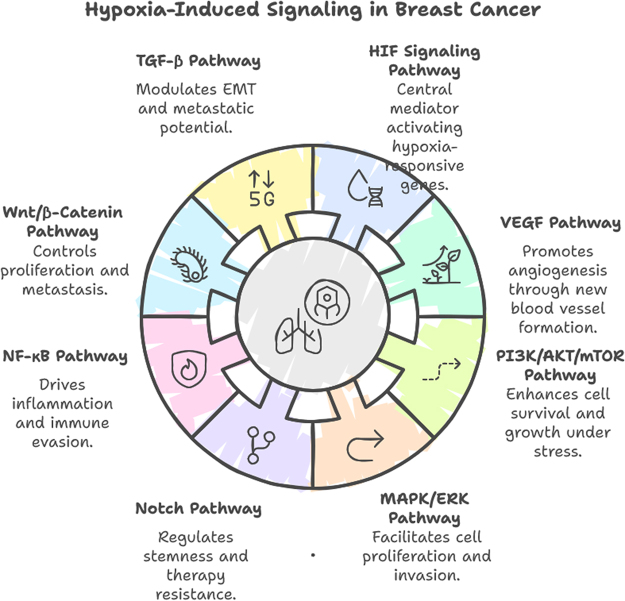
Hypoxia-induced signaling in breast cancer.

Hypoxia also exerts its effects through epigenetic modifications, including DNA methylation, histone modifications, and non-coding RNA regulation. HIF-1α recruits epigenetic modifiers to regulate the transcription of hypoxia-responsive genes. Hypoxia-induced microRNAs (e.g., miR-210) play critical roles in modulating angiogenesis, metabolism, and cell survival, further highlighting the complexity of hypoxia-driven signaling networks^[24]^ (Figure 2).

### Hypoxia across breast cancer subtypes: molecular signatures

Breast cancer is a highly heterogeneous disease comprising multiple molecular subtypes, each with distinct biological behaviors, prognoses, and responses to therapy. These subtypes – commonly categorized as Luminal A, Luminal B, HER2-enriched, and Triple-Negative/Basal-like – differ not only in hormone receptor status and proliferative indices but also in their microenvironmental characteristics, particularly the degree and consequences of hypoxia. Emerging evidence indicates that the extent of hypoxia and the transcriptional response it elicits vary significantly across subtypes, shaping tumor progression and treatment resistance in subtype-specific ways.

### Luminal A and luminal B subtypes

Luminal A and B breast cancers are characterized by the expression of estrogen and/or progesterone receptors, with Luminal B exhibiting higher proliferative activity and more aggressive features. Generally, Luminal A tumors exhibit the lowest levels of hypoxia, attributed to their slower growth rates and better-structured vasculature. However, Luminal B tumors, particularly those with high Ki-67 expression, are more susceptible to developing hypoxic zones due to increased metabolic demand and relative vascular insufficiency. In these luminal subtypes, HIF-1α remains the central transcription factor mediating oxygen-sensing responses. In Luminal B tumors, increased HIF-1α activity correlates with upregulation of VEGF, promoting angiogenesis, and enhanced expression of carbonic anhydrase IX (CAIX), facilitating pH regulation in an acidic, hypoxic microenvironment. Interestingly, in hormone receptor-positive tumors, HIF-1α can cross-talk with estrogen receptor (ER) signaling, modulating genes involved in metabolism and cell proliferation. This interaction may contribute to endocrine resistance, particularly in Luminal B tumors under chronic hypoxia^[1,2]^.

### HER2-enriched subtype

HER2-positive breast cancers are defined by the overexpression of the HER2 receptor tyrosine kinase, often accompanied by aggressive clinical behavior. In this subtype, hypoxia frequently coexists with rapid tumor expansion, leading to the activation of both HIF-1α and HIF-2α. These transcription factors drive the upregulation of glucose transporter 1 (GLUT1) and lactate dehydrogenase A (LDHA), supporting a glycolytic phenotype and promoting survival in low-oxygen environments. Molecular profiling of HER2-enriched tumors under hypoxic stress reveals activation of PI3K/Akt/mTOR signaling, further enhancing HIF stabilization and downstream gene expression. Additionally, hypoxia can induce hypoxia-mediated HER2 signaling feedback loops, reinforcing tumor cell proliferation and potentially reducing sensitivity to anti-HER2 therapies such as trastuzumab^[4]^.

### Triple-negative/basal-like breast cancer (TNBC/BLBC)

Among all breast cancer subtypes, triple-negative and basal-like tumors exhibit the highest degree of hypoxia. This is largely due to their aggressive nature, high mitotic index, and poor vascular organization. These tumors are notorious for their enriched hypoxic gene signatures, including marked overexpression of HIF-1α, HIF-2α, CAIX, VEGF, and multiple glycolytic enzymes. What sets TNBC apart is the dominance of hypoxia-associated stemness and epithelial-mesenchymal transition (EMT) programs. Under hypoxic conditions, TNBC cells activate transcription factors such as Snail, Twist, and ZEB1, which drive EMT and enhance metastatic potential. Hypoxia also sustains breast cancer stem cell (BCSC) populations via Notch and Wnt signaling, conferring resistance to chemotherapy and promoting tumor relapse. Furthermore, TNBC tumors frequently demonstrate immune evasive phenotypes under hypoxia, with increased expression of PD-L1 and recruitment of immunosuppressive MDSCs. The presence of a distinct hypoxia gene signature in TNBC, often including genes like LOX, ANGPTL4, and ENO1, has been linked to poor prognosis and aggressive clinical outcomes^[5]^.

### Implications of hypoxia heterogeneity

The heterogeneity of hypoxia responses across breast cancer subtypes not only reflects intrinsic biological diversity but also underscores the need for subtype-specific therapeutic strategies. While anti-hypoxia therapies may offer limited benefit in Luminal A cancers, they hold considerable promise in TNBC, where hypoxia is both extensive and mechanistically pivotal. Moreover, understanding these differential hypoxic responses may guide the development of hypoxia-related biomarkers for prognosis and treatment selection.

### Hypoxia-Induced metabolic reprogramming and its impact on breast cancer therapy response

In the dynamic microenvironment of breast cancer, hypoxia triggers profound metabolic reprogramming, most notably a shift from oxidative phosphorylation to aerobic glycolysis, even in the presence of sufficient glucose and oxygen – a phenomenon widely recognized as the Warburg effect. This hypoxia-driven glycolytic switch is not merely a metabolic adaptation for energy production under oxygen deprivation; rather, it orchestrates a survival strategy that enhances tumor resilience and contributes significantly to therapeutic resistance, particularly against chemotherapy and targeted therapies^[5]^. At the molecular level, HIF-1α is the key regulator of this reprogramming. Stabilized under low oxygen conditions, HIF-1α upregulates the expression of glucose transporter 1 (GLUT1), hexokinase 2 (HK2), lactate dehydrogenase A (LDHA), and pyruvate dehydrogenase kinase 1 (PDK1). Together, these effectors promote increased glucose uptake and lactate production while suppressing mitochondrial oxidative phosphorylation, thereby rerouting cellular metabolism towards glycolysis^[6]^. This metabolic shift has several downstream implications that blunt the efficacy of chemotherapy and targeted treatments. Firstly, enhanced glycolysis leads to increased acidification of the tumor microenvironment, driven by lactate accumulation. The acidic milieu impairs drug penetration and reduces the intracellular concentration of chemotherapeutic agents such as doxorubicin, cyclophosphamide, and paclitaxel – key components of breast cancer regimens. Moreover, acidic pH alters the ionization of weakly basic drugs, reducing their ability to cross cell membranes and accumulate in cancer cells effectively^[7]^.

Secondly, hypoxia-induced glycolysis supports anti-apoptotic signaling and drug detoxification mechanisms. For instance, the upregulation of LDHA and pyruvate metabolism intermediates under hypoxia can buffer oxidative stress and reduce reactive oxygen species (ROS) formation, a key mechanism through which many chemotherapeutics induce cell death. As a result, tumor cells under hypoxic and glycolytic stress become more tolerant to DNA-damaging agents^[8]^. In HER2-positive breast cancers, targeted therapies such as trastuzumab and lapatinib rely on the disruption of HER2-driven proliferative signaling. However, under hypoxic conditions, metabolic reprogramming can activate compensatory pathways, such as the PI3K/Akt/mTOR cascade, which further stabilize HIF-1α and sustain glycolysis. This positive feedback loop fosters resistance by preserving cellular energy production and survival independent of HER2 blockade^[9]^. The situation is even more pronounced in triple-negative breast cancer (TNBC), which lacks hormone receptors and HER2 expression. These tumors, already intrinsically aggressive and therapy-resistant, exhibit high baseline glycolytic activity and are particularly adept at surviving under hypoxic stress. In TNBC, hypoxia promotes the expression of glycolytic enzymes and EMT-related genes, enhancing not only resistance to chemotherapy but also the likelihood of recurrence and metastasis^[5]^. Furthermore, glycolytic reprogramming supports the maintenance of breast cancer stem-like cells, which are notoriously resistant to standard therapies. These cells, enriched in hypoxic niches, utilize glycolysis for energy and redox balance, allowing them to persist post-treatment and drive tumor regrowth^[10]^. Recognizing the centrality of hypoxia-driven glycolysis in therapeutic resistance has prompted interest in targeting metabolic pathways as an adjunct to existing treatments. Inhibitors of LDHA, GLUT1, and PDK1, as well as agents that normalize tumor vasculature and reoxygenate the microenvironment, are under investigation for their ability to sensitize tumors to chemotherapy and targeted agents^[11]^.

### Anti-angiogenic therapies: modulating hypoxia and breast cancer progression

The vascular network of a tumor is both its lifeline and its liability. Rapidly growing breast tumors often outpace their blood supply, resulting in poorly organized, leaky, and inefficient vasculature. This dysfunctional network contributes to regional hypoxia, a condition that fuels tumor aggression, immune escape, and therapeutic resistance. In this context, anti-angiogenic therapies, designed to inhibit the formation of new blood vessels, have emerged as a double-edged sword in the management of breast cancer^[12]^. At the core of anti-angiogenic therapy is the inhibition of VEGF, a potent angiogenic driver often overexpressed under hypoxic conditions. Agents such as bevacizumab, a monoclonal antibody against VEGF-A, aim to starve tumors of their blood supply, thereby impeding growth. While the rationale appears straightforward – cut off the fuel line to halt the fire – clinical and experimental evidence reveals a more complex interplay between angiogenesis, hypoxia, and tumor behavior^[13]^. Initially, anti-angiogenic therapy can induce a “vascular normalization” window. During this transient phase, abnormal tumor vessels become more structured, less permeable, and better perfused. Paradoxically, this normalization improves oxygenation and reduces hypoxia, thereby enhancing the delivery and effectiveness of chemotherapeutic agents and reducing the hypoxia-mediated expression of pro-survival and metastatic genes. Some studies suggest that this phase can sensitize tumors to chemotherapy and radiation, particularly in subtypes like HER2-positive or Luminal B breast cancers^[8]^.

However, prolonged or high-dose anti-angiogenic therapy may intensify hypoxia, especially in inherently hypoxic subtypes like triple-negative breast cancer (TNBC). By pruning existing vessels without adequate normalization, therapy can exacerbate oxygen deprivation. The tumor, now in a state of oxygen starvation, activates compensatory survival pathways – most notably HIF-1α. HIF-1α upregulates alternative angiogenic factors (e.g., FGF, PDGF) and promotes metabolic reprogramming toward glycolysis, leading to tumor adaptation and resistance^[14]^. More concerning is the observation that heightened hypoxia after aggressive anti-angiogenic therapy can enhance epithelial-mesenchymal transition (EMT) and stemness – hallmarks of metastasis and recurrence. In TNBC, where baseline hypoxia is already substantial, anti-angiogenic treatment has been shown to inadvertently increase the invasiveness of cancer cells, sometimes shifting the pattern of progression from local growth to distant dissemination^[15]^. Moreover, anti-angiogenic therapy affects immune cell dynamics within the tumor microenvironment. Hypoxia upregulation of PD-L1 and recruitment of MDSCs and regulatory T cells (Tregs) contributes to immune suppression, diminishing the efficacy of immune surveillance and immunotherapies. Thus, the immunological landscape of breast tumors may worsen under prolonged hypoxia induced by such treatments^[16]^.

### The Immunosuppressive face of hypoxia: implications for immune checkpoint inhibitor therapy in breast cancer

In the tumor microenvironment of breast cancer, hypoxia is more than just a lack of oxygen – it is a powerful architect of immune suppression. As oxygen levels dwindle within poorly perfused tumor regions, a cascade of molecular adaptations reshapes immune cell behavior, metabolic activity, and intercellular signaling. These changes collectively weaken anti-tumor immunity, creating a sanctuary where cancer cells can thrive despite therapeutic intervention. Of particular concern is the impact of hypoxia on the efficacy of immune checkpoint inhibitors (ICIs) – a class of therapies that relies on the reactivation of T cells to eliminate tumor cells^[17]^. At the center of this immunosuppressive storm is HIF-1α, the master regulator of cellular response to low oxygen. Stabilized under hypoxic conditions, HIF-1α drives transcriptional programs that profoundly alter immune cell function and tumor-immune interactions. One of the key mechanisms involves the upregulation of programmed death-ligand 1 (PD-L1) on tumor cells and some immune cells. PD-L1 binds to PD-1 receptors on activated T cells, triggering an inhibitory signal that dampens T cell proliferation, cytokine production, and cytolytic activity – essentially putting the brakes on the immune attack^[18]^. While PD-1/PD-L1 inhibitors aim to disrupt this interaction and restore T cell function, hypoxia complicates this therapeutic pathway. Elevated PD-L1 expression under hypoxic stress may initially seem to favor checkpoint blockade. However, hypoxia concurrently orchestrates a broader immune evasion strategy that diminishes the overall efficacy of ICIs. For instance, HIF-1α signaling promotes the recruitment and expansion of immunosuppressive cell types, such as regulatory T cells (Tregs) and MDSCs. These cells actively suppress effector T cell activity, either by releasing inhibitory cytokines like IL-10 and TGF-β or by direct cell-to-cell contact mechanisms^[19]^.

In addition, hypoxia affects antigen presentation by downregulating major histocompatibility complex class I (MHC-I) molecules on tumor cells, making them less visible to cytotoxic CD8+ T cells. This “immune invisibility” further undermines the potential for immune checkpoint inhibitors to trigger a meaningful anti-tumor response. Dendritic cells (DCs), which play a pivotal role in T cell priming, also suffer under hypoxic conditions – exhibiting impaired maturation, antigen uptake, and costimulatory signaling^[20]^. Moreover, the metabolic reprogramming induced by hypoxia creates a hostile environment for immune cells. Hypoxia pushes tumor cells toward aerobic glycolysis, resulting in lactic acid accumulation and acidification of the tumor milieu. High lactate levels inhibit T cell motility and function, while simultaneously promoting the polarization of macrophages toward a tumor-supportive M2 phenotype, further skewing the immune balance in favor of immune tolerance rather than attack^[21]^. In breast cancer subtypes, particularly triple-negative breast cancer (TNBC), which has emerged as a candidate for immunotherapy, the heterogeneous hypoxic landscape presents additional challenges. While TNBC may express higher levels of PD-L1, the accompanying hypoxic microenvironment often limits the durability and depth of responses to ICIs. Clinical trials have shown modest responses to checkpoint blockade in breast cancer compared to other cancers, partly due to these hypoxia-driven barriers^[22]^. Emerging strategies are attempting to overcome hypoxia-induced immune resistance. Hypoxia-targeted agents, HIF-1α inhibitors, and metabolic modulators such as lactate dehydrogenase (LDH) inhibitors are being studied for their potential to recondition the tumor microenvironment. The goal is to transform the “cold,” immunosuppressed tumor landscape into a “hot,” inflamed, and immunologically responsive state where immune checkpoint inhibitors can exert their full potential^[23]^.

### Biomarkers of tumor hypoxia in breast cancer and their role in clinical decision-making

In the labyrinth of breast cancer biology, hypoxia emerges as a stealthy force – unseen by the naked eye but pivotal in shaping tumor behavior, therapy resistance, and patient outcomes. The ability to reliably quantify tumor hypoxia has become an urgent need, not only for understanding prognosis but also for tailoring therapeutic strategies. To meet this challenge, researchers and clinicians have turned to a range of biomarkers – molecular, imaging-based, and physiologic tools – that offer a window into the oxygen-starved microenvironment of breast tumors.

### Molecular biomarkers

At the molecular level, HIF-1α stands as the master regulator and a frequently studied surrogate marker of hypoxia. Stabilized in low oxygen conditions, HIF-1α drives the transcription of numerous genes that collectively signal a hypoxic tumor milieu. Elevated expression of HIF-1α in breast cancer tissue – especially in triple-negative and HER2-positive subtypes – has been correlated with poor prognosis, enhanced angiogenesis, and resistance to therapy. Downstream targets of HIF-1α, such as carbonic anhydrase IX (CAIX), glucose transporter 1 (GLUT1), and VEGF, have emerged as robust markers for hypoxia-induced adaptations. CAIX, in particular, has been extensively validated in breast cancer specimens, where its membrane localization and relative stability make it an attractive candidate for immunohistochemical evaluation in biopsy samples. Elevated CAIX expression often signifies a more aggressive tumor phenotype and correlates with radioresistance and chemotherapy failure^[24]^.

### Imaging biomarkers

To complement molecular insights, non-invasive imaging technologies allow for spatial and temporal mapping of hypoxia in vivo. Techniques such as positron emission tomography (PET) using radiotracers like [^18^F]-fluoromisonidazole (FMISO) or [^64^Cu]-ATSM enable visualization of hypoxic zones within tumors. These tracers selectively accumulate in hypoxic cells, offering a powerful way to monitor hypoxia dynamics during treatment. Functional magnetic resonance imaging (fMRI), including blood oxygen level-dependent (BOLD) MRI and dynamic contrast-enhanced (DCE) MRI, also provides indirect measurements of tumor oxygenation. While technically demanding, these modalities are gradually being refined for clinical use and may soon help predict treatment response or monitor therapeutic modulation of hypoxia^[25]^.

### Blood-based biomarkers

Although still in development, circulating biomarkers are gaining traction for their potential to reflect tumor hypoxia through a simple blood draw. These include extracellular vesicles, microRNAs (e.g., miR-210), and soluble CAIX, which may offer insight into tumor oxygen status with less invasive sampling. While promising, further validation is needed before these can be adopted into routine practice^[26]^.

### Integrating hypoxia biomarkers into clinical decision-making

The true value of hypoxia biomarkers lies not just in measurement, but in clinical application. When integrated thoughtfully, these markers can refine several aspects of breast cancer management:

- **Risk Stratification and Prognosis**: High levels of HIF-1α or CAIX in tumor tissue can signal poor prognosis, informing clinicians about likely resistance patterns and recurrence risk – particularly in aggressive subtypes like basal-like or triple-negative breast cancer.
- **Therapy Selection**: Tumors with strong hypoxia signatures may benefit from hypoxia-targeted therapies (e.g., HIF-1α inhibitors, CAIX inhibitors) or altered radiation schedules designed to overcome hypoxia-induced resistance. Additionally, knowing the hypoxic burden can guide the use of metabolic modulators or vascular normalization strategies in combination therapies.
- **Monitoring Treatment Response**: Repeated imaging with hypoxia-specific tracers during chemotherapy or anti-angiogenic therapy can provide real-time feedback on therapeutic effectiveness, allowing clinicians to adapt strategies dynamically.
- **Predicting Immunotherapy Response**: Given the immunosuppressive effects of hypoxia, assessing hypoxia status may help predict checkpoint inhibitor efficacy and identify patients who might benefit from combination therapies that reverse hypoxic suppression^[27]^.

### Hypoxia as a catalyst for EMT and metastasis in breast cancer

In the complex symphony of breast cancer progression, hypoxia plays a haunting note – subtle yet deeply transformative. As tumors expand beyond their vascular supply, oxygen availability dwindles, creating hypoxic zones that trigger a cascade of adaptive responses. Among the most consequential of these responses is the promotion of EMT – a cellular metamorphosis that endows tumor cells with invasive and migratory capabilities, effectively priming them for metastatic escape^[5]^. EMT is a biological process in which epithelial cells lose their polarity and adhesion properties, acquiring a mesenchymal phenotype characterized by enhanced motility, invasiveness, and resistance to apoptosis. In breast cancer, particularly in aggressive subtypes such as triple-negative and basal-like tumors, hypoxia has been recognized as a powerful driver of EMT^[10]^. The molecular engine behind this transition is once again HIF-1α. Stabilized in the absence of oxygen, HIF-1α orchestrates the expression of a suite of transcription factors – Snail, Slug, Twist, and ZEB1/2 – that actively repress epithelial markers such as E-cadherin and promote mesenchymal markers like vimentin and N-cadherin. The downregulation of E-cadherin, a key component of cell-cell adhesion, facilitates the detachment of tumor cells from the primary mass, while the upregulation of mesenchymal genes enhances cytoskeletal reorganization and migratory behavior^[11]^.

Moreover, hypoxia amplifies EMT by remodeling the tumor microenvironment. It stimulates the secretion of matrix metalloproteinases (MMPs), particularly MMP-2 and MMP-9, which degrade extracellular matrix components and create pathways for invasion. It also promotes the recruitment of stromal cells – such as cancer-associated fibroblasts and tumor-associated macrophages – which further support EMT through paracrine signaling and matrix restructuring^[12]^. As EMT progresses, tumor cells gain the ability to intravasate into blood and lymphatic vessels, navigating through endothelial barriers with newfound plasticity. These disseminated cells, often referred to as circulating tumor cells (CTCs), carry mesenchymal traits that not only enhance their survival in circulation but also prepare them for colonization at distant metastatic sites^[13]^. Interestingly, hypoxia does not only initiate EMT but also supports mesenchymal-to-epithelial transition (MET) at secondary sites. This reversible plasticity enables disseminated cells to reacquire epithelial features necessary for proliferation and establishment of metastatic lesions, such as those commonly seen in the lungs, liver, and bones of breast cancer patients^[26]^. Clinical evidence supports this narrative. Tumors with hypoxic gene signatures and high EMT marker expression are frequently associated with advanced stage, lymph node involvement, and poor prognosis. Furthermore, hypoxia-driven EMT has been linked to resistance to conventional therapies, including chemotherapy and radiotherapy, complicating treatment and contributing to disease recurrence^[27]^.

### Hypoxia-driven signaling pathways and therapeutic resistance in breast cancer

In the evolving narrative of breast cancer, hypoxia stands out as a formidable antagonist – fueling not just tumor progression, but also resistance to therapy, a key challenge in modern oncology. While its role in altering tumor metabolism and microenvironment is well recognized, the true depth of hypoxia’s impact lies in its ability to reprogram intracellular signaling pathways. These rewired circuits enable breast cancer cells to resist cytotoxic pressures from chemotherapy, radiotherapy, endocrine therapy, and targeted agents, ultimately leading to treatment failure and disease relapse. Several key signaling pathways emerge as central players in this hypoxia-mediated resistance, each offering unique insights and potential targets for therapeutic intervention^[28]^.

1. **HIF-1α Pathway**

At the core of hypoxia-induced resistance lies the HIF-1α pathway. Under normoxic conditions, HIF-1α is rapidly degraded. But in the oxygen-starved tumor core, it stabilizes and translocates to the nucleus, activating transcription of genes that promote angiogenesis (e.g., VEGF), glycolysis (e.g., GLUT1), and cell survival (e.g., BCL2). This adaptation facilitates tumor cell survival under harsh conditions and creates a hostile environment for therapeutic agents. For instance, by upregulating MDR1 (multi-drug resistance gene), HIF-1α can increase drug efflux from cells, reducing intracellular concentrations of chemotherapeutic agents. Small-molecule inhibitors of HIF-1α (e.g., PX-478) and strategies aimed at degrading HIF-1α are under investigation, though their clinical translation remains a work in progress due to toxicity and specificity challenges^[29]^.

**2. PI3K/AKT/mTOR Pathway**

Another major player in hypoxia-driven resistance is the PI3K/AKT/mTOR pathway. Hypoxia activates this signaling cascade through both HIF-1α-dependent and independent mechanisms, promoting cell proliferation, survival, and metabolic reprogramming. In estrogen receptor-positive (ER+) breast cancers, activation of this pathway under hypoxia contributes to endocrine therapy resistance, while in HER2+ subtypes, it undermines the efficacy of anti-HER2 agents like trastuzumab. Clinically approved inhibitors such as everolimus (an mTOR inhibitor) and investigational dual PI3K/mTOR inhibitors have shown promise in overcoming resistance when used in combination with endocrine or targeted therapies^[30]^.

**3. Notch and Wnt/β-Catenin Pathways**

Hypoxia also stimulates the Notch and Wnt/β-catenin signaling pathways, both of which are implicated in maintaining cancer stem cell (CSC) populations. These cells, though few in number, are notoriously resistant to chemotherapy and radiation and are believed to drive tumor relapse. Hypoxia-induced HIF-1α upregulates Notch1 and its ligand Jagged1, while also enhancing β-catenin stabilization, collectively supporting the self-renewal and survival of CSCs. Gamma-secretase inhibitors (GSIs) that block Notch signaling and Wnt/β-catenin inhibitors are under investigation in preclinical and early-phase clinical trials to reduce CSC populations and sensitize tumors to therapy^[31]^.

**4. NF-κB Pathway**

Hypoxia activates nuclear factor kappa-light-chain-enhancer of activated B cells (NF-κB), a key transcription factor in inflammation and survival signaling. NF-κB activation leads to upregulation of anti-apoptotic proteins such as BCL-XL and XIAP, enhancing resistance to chemotherapy and radiation. It also contributes to a pro-inflammatory, immunosuppressive tumor microenvironment, further shielding tumor cells from immune surveillance. Inhibitors of IKK (IκB kinase), which regulate NF-κB activation, have shown therapeutic potential in preclinical models but require careful optimization due to their role in normal immune function^[32]^.

**5. MAPK/ERK Pathway**

The MAPK/ERK signaling pathway, often activated in hypoxia, plays a role in cell cycle progression, survival, and angiogenesis. Its crosstalk with HIF-1α signaling potentiates the expression of hypoxia-responsive genes, enhancing resistance to both conventional therapies and newer agents. MEK inhibitors have been explored in clinical trials for breast cancer, particularly in combination with chemotherapy or PI3K inhibitors, to overcome compensatory survival signaling^[33]^.

### Hypoxia, stromal allies, and immune accomplices in breast cancer progression

Within the crowded theater of breast cancer biology, hypoxia acts not only as a stressor but as a powerful director – shaping the behavior of surrounding actors, especially stromal and immune cells. The tumor microenvironment (TME), already a complex ecosystem of malignant and non-malignant components, becomes further distorted under the grip of low oxygen tension. In this altered landscape, cellular roles are redefined, communications reshuffled, and a subtle alliance is formed to advance tumor growth, invasion, and immune evasion^[34]^.

### Hypoxia and stromal cells

In response to hypoxia, stromal cells, particularly cancer-associated fibroblasts (CAFs), undergo profound reprogramming. Hypoxia transforms these fibroblasts into active accomplices that secrete pro-tumorigenic cytokines (e.g., TGF-β, IL-6) and growth factors (e.g., VEGF, PDGF), which promote angiogenesis, extracellular matrix (ECM) remodeling, and EMT in nearby cancer cells. Hypoxia also upregulates matrix metalloproteinases (MMPs) in CAFs, particularly MMP-2 and MMP-9, facilitating degradation of the ECM and paving paths for tumor cell invasion. Simultaneously, HIF-1α signaling promotes the expression of lysyl oxidase (LOX), an enzyme that stiffens the ECM, enhancing the migratory potential of tumor cells and supporting metastatic dissemination. Moreover, stromal cells in hypoxic zones exhibit increased secretion of exosomes loaded with pro-survival, EMT-inducing, and immune-modulating cargo. These vesicles mediate long-distance crosstalk, influencing not just local tumor dynamics but also preparing distant sites – so-called pre-metastatic niches – for colonization^[35]^.

### Immune cells under hypoxia

The immune compartment within the hypoxic breast tumor microenvironment undergoes a dramatic shift from surveillance to suppression. Hypoxia skews the recruitment and function of various immune cell subsets in favor of immune escape and chronic inflammation – key hallmarks of cancer progression.

1. **Tumor-Associated Macrophages (TAMs)**

Under normoxia, macrophages can exhibit tumor-suppressive (M1-like) properties. However, in hypoxic zones, macrophages are polarized toward an M2-like phenotype, driven by HIF-1α and HIF-2α. These M2-TAMs produce IL-10, TGF-β, and VEGF, all of which suppress cytotoxic T-cell activity and promote angiogenesis and tissue remodeling. They also facilitate lymphangiogenesis and support metastasis to lymph nodes and distant organs.

**2. Myeloid-Derived Suppressor Cells (MDSCs)**

Hypoxia enhances the recruitment and expansion of MDSCs, which inhibit T-cell proliferation and function via production of arginase-1, reactive oxygen species (ROS), and nitric oxide (NO). These immunosuppressive cells further obstruct antitumor immunity and can impair the efficacy of immunotherapy^[36]^.

**3. Regulatory T Cells (Tregs)**

The hypoxic milieu favors the recruitment and survival of Tregs, which suppress effector T-cell responses through the release of IL-10 and TGF-β. HIF-1α directly induces the transcription of FoxP3, a master regulator of Treg function, cementing their suppressive role in the TME.

**4. Dendritic Cells (DCs)**

Antigen-presenting dendritic cells lose their functional competency in hypoxic zones. Instead of priming cytotoxic T-cell responses, these hypoxia-impaired DCs exhibit a tolerogenic phenotype, further blunting adaptive immunity and aiding immune evasion^[37]^.

## Crosstalk and feedback

What makes the hypoxic TME even more formidable is the feedback loop it establishes. Stromal and immune cells, once reprogrammed by hypoxia, in turn secrete factors that sustain and intensify the hypoxic condition. For example, VEGF secreted by CAFs and TAMs induces chaotic, leaky blood vessels that fail to adequately perfuse the tumor, perpetuating oxygen deprivation. This vicious cycle of hypoxia and cellular reprogramming ensures a stable niche that fosters aggressive, therapy-resistant tumors^[38]^.

### Emerging precision medicine approaches in breast cancer: improving treatment efficacy and reducing adverse effects

Breast cancer remains one of the most common cancers worldwide, with significant variability in how patients respond to treatment. Traditional treatment approaches, which include surgery, chemotherapy, radiation, and hormone therapy, have provided considerable advancements in patient survival. However, these treatments often fail to account for the underlying genetic, molecular, and environmental factors that contribute to tumor heterogeneity. In this context, precision medicine has emerged as a transformative approach in oncology, particularly in breast cancer, by tailoring treatments based on individual patient profiles. This personalized approach promises to enhance treatment efficacy and reduce adverse effects by targeting the unique characteristics of each patient’s tumor^[39]^.

### Understanding precision medicine in breast cancer

Precision medicine, also known as personalized medicine, involves the use of genetic, molecular, and environmental data to guide treatment decisions. In breast cancer, this approach encompasses a broad spectrum of strategies, including genomic profiling, targeted therapies, immune therapies, and the identification of biomarkers that can predict treatment response. The primary goal is to match patients with the most appropriate therapies based on the specific molecular characteristics of their cancer, thereby optimizing outcomes and minimizing unnecessary side effects^[40]^.

### Improving treatment efficacy through targeted therapies

Targeted therapies are one of the cornerstones of precision medicine. Unlike traditional chemotherapy, which indiscriminately affects both cancerous and healthy cells, targeted therapies focus on specific molecular targets involved in the growth and survival of cancer cells. These therapies have demonstrated superior efficacy in treating certain subtypes of breast cancer, particularly those driven by specific genetic mutations or overexpressed proteins^[41]^.

1. **HER2-Positive Breast Cancer**

One of the most notable success stories of precision medicine in breast cancer is the treatment of HER2-positive breast cancer, a subtype characterized by the overexpression of the human epidermal growth factor receptor 2 (HER2). Traditionally, this subtype was associated with poor prognosis and aggressive disease. However, the development of HER2-targeted therapies, such as trastuzumab (Herceptin), has significantly improved outcomes for patients with HER2-positive breast cancer. Trastuzumab works by binding to the HER2 receptor, inhibiting its signaling pathway, and triggering immune responses that target the cancer cells. This targeted approach has been shown to reduce tumor size, prevent recurrence, and improve overall survival in patients with HER2-positive breast cancer^[42]^.

**2. Hormone Receptor-Positive Breast Cancer**

Hormone receptor-positive breast cancers (HR-positive), which express estrogen and/or progesterone receptors, are another example of precision medicine in action. These tumors are sensitive to hormones, and therapies that block hormone signaling, such as selective estrogen receptor modulators (SERMs) like tamoxifen or aromatase inhibitors like letrozole, have been a cornerstone of treatment for HR-positive breast cancer. Recently, the development of CDK4/6 inhibitors (e.g., palbociclib) has further improved outcomes by blocking cell cycle progression in HR-positive breast cancer cells. These treatments, when combined with hormone therapy, offer a more tailored and effective approach, improving progression-free survival and overall survival while reducing the need for aggressive chemotherapy^[43]^.

**3. BRCA-Mutated Breast Cancer**

BRCA1 and BRCA2 mutations significantly increase the risk of developing breast cancer and are associated with a more aggressive disease course. Precision medicine has revolutionized the treatment of BRCA-mutated breast cancers with the introduction of poly(ADP-ribose) polymerase (PARP) inhibitors, such as olaparib. PARP inhibitors exploit the DNA repair defects caused by BRCA mutations, leading to cancer cell death. These therapies have shown significant efficacy in patients with BRCA mutations, providing an alternative to traditional chemotherapy and improving survival rates in this subgroup^[44]^.

### Reducing adverse effects with precision medicine

A major limitation of conventional cancer treatments, particularly chemotherapy, is their toxicity, which affects not only the tumor cells but also healthy tissues. Precision medicine offers a promising solution by reducing the risk of side effects through the identification of molecular markers that predict individual responses to treatment.

**1. Genomic Profiling and Predictive Biomarkers**

Genomic profiling allows for the identification of specific mutations, gene expressions, and tumor microenvironment characteristics that may influence treatment response. By analyzing a patient’s tumor DNA, doctors can identify predictive biomarkers that can help guide treatment decisions and reduce the likelihood of adverse reactions. For example, patients with certain genetic variants might experience severe toxicity when treated with specific chemotherapies. By identifying these variants in advance, clinicians can avoid harmful treatments or adjust dosages to minimize side effects. For example, the CYP2D6 gene, which encodes an enzyme responsible for metabolizing tamoxifen, can influence how well patients respond to this drug. Patients with certain genetic variations may not convert tamoxifen to its active form as effectively, leading to reduced efficacy. Genomic profiling allows clinicians to identify these patients early and adjust the treatment strategy accordingly, such as switching to an alternative therapy^[45]^.

**2. Immune Checkpoint Inhibitors**

Immune checkpoint inhibitors, such as pembrolizumab, are a new class of therapies that harness the body’s immune system to fight cancer. By targeting immune checkpoint proteins like PD-1/PD-L1, these inhibitors can enhance the immune system’s ability to recognize and destroy cancer cells. Precision medicine plays a critical role in identifying patients who are most likely to benefit from these treatments. Tumors that express high levels of PD-L1, for instance, are more likely to respond to immune checkpoint inhibitors, allowing for a more targeted approach and reducing the risk of ineffective treatments and associated side effects^[46]^.

**3. Pharmacogenomics and Dose Optimization**

Pharmacogenomics is the study of how genetic variations influence drug responses. By incorporating pharmacogenomic testing into clinical practice, clinicians can tailor the choice and dosage of medications to an individual’s genetic profile, thereby optimizing drug efficacy and minimizing adverse reactions. For example, the chemotherapy drug 5-fluorouracil (5-FU) is known to cause severe toxicity in patients with a specific genetic variant of the DPD (dihydropyrimidine dehydrogenase) enzyme. Pharmacogenomic testing can identify these patients in advance, enabling dose adjustments or alternative therapies to be considered, reducing the risk of life-threatening side effects^[47]^.

### The role of liquid biopsy in monitoring treatment response

Liquid biopsy, which involves analyzing blood samples for tumor-derived genetic material, offers a non-invasive method for monitoring treatment response and detecting minimal residual disease. This approach can help assess how well a patient is responding to a given therapy and provide early indications of relapse. By tracking changes in circulating tumor DNA (ctDNA) or other biomarkers, clinicians can adjust treatment plans in real-time, further optimizing treatment efficacy and minimizing unnecessary side effects^[48]^.

### The role of artificial intelligence in breast cancer diagnosis and treatment planning: current impact and limitations

Breast cancer, as one of the most prevalent cancers globally, demands precision in its diagnosis and treatment planning. Over the years, advancements in technology have revolutionized the way healthcare professionals approach the management of this disease. Among these innovations, Artificial Intelligence (AI) has emerged as a powerful tool in both diagnosing breast cancer and developing personalized treatment plans. AI-driven approaches leverage vast amounts of data to support clinicians, improve the accuracy of diagnoses, and optimize treatment regimens. While AI is showing great promise, challenges remain in fully realizing its potential in the clinical setting. This narrative explores the impact of AI on breast cancer diagnosis and treatment planning and addresses the current limitations of AI-driven approaches^[49]^.

### AI in breast cancer diagnosis

Early detection of breast cancer is crucial to improving patient outcomes. Traditional diagnostic methods, such as mammography, ultrasound, and biopsy, have been foundational in breast cancer detection. However, these techniques have inherent limitations, including operator dependence, variability in interpretation, and potential for false positives or negatives. AI has the potential to overcome many of these challenges, providing more accurate and efficient methods for detecting breast cancer.

1. **AI in Imaging: Improving Accuracy and Reducing Human Error**

One of the most prominent applications of AI in breast cancer diagnosis is in the interpretation of medical imaging, particularly mammograms. AI algorithms, particularly deep learning models, have shown remarkable accuracy in detecting abnormal growths, such as tumors, cysts, or calcifications that may indicate the presence of breast cancer. By training on vast datasets of annotated mammogram images, AI models can learn to identify subtle patterns that might be missed by human radiologists. For example, AI systems like Google’s DeepMind have demonstrated the ability to outperform radiologists in identifying breast cancer in mammograms, with some models achieving higher sensitivity and specificity than human counterparts. AI’s ability to process and analyze large volumes of imaging data allows for quicker and more consistent results, reducing the likelihood of human error^[50]^.

**2. AI in Risk Assessment: Predicting Cancer Development**

AI has also been used to assess a patient’s risk of developing breast cancer. By analyzing a combination of imaging data, genetic information, and patient medical history, AI models can provide a more nuanced understanding of an individual’s risk factors. Machine learning algorithms can identify complex relationships between genetic mutations (e.g., BRCA1/2) and environmental factors, improving predictive models for identifying those at high risk. This allows for more proactive screening, personalized surveillance, and preventive strategies tailored to individual patients^[51]^.

**3. AI in Histopathological Analysis**

Another critical area where AI is being employed is in histopathology, where tissue samples are analyzed for cancerous cells. AI-powered tools can analyze digital slides of biopsy specimens with great precision, identifying malignant cells and providing a more detailed tumor grade. AI can also assess the tumor’s molecular profile by examining genetic and protein expressions, further guiding treatment choices such as whether a patient would benefit from targeted therapies like HER2 inhibitors or hormone blockers. This enables more accurate staging and prognostic evaluation, critical components in determining the most appropriate treatment plan^[52]^.

### AI in breast cancer treatment planning

Once a breast cancer diagnosis is confirmed, treatment planning involves selecting the most appropriate course of action based on the tumor’s type, size, location, and the patient’s overall health. AI has shown immense promise in improving treatment planning by offering personalized recommendations and optimizing therapeutic strategies.

**1. Personalized Medicine and AI**

Precision medicine relies on understanding the genetic and molecular characteristics of a patient’s tumor. AI is playing a pivotal role in this aspect by analyzing genomic data to identify mutations and other biomarkers that might influence treatment responses. For instance, AI algorithms can rapidly analyze whole-genome sequencing data to identify mutations like BRCA1/2 or PIK3CA that can inform treatment options, such as PARP inhibitors or PI3K inhibitors. AI can integrate data from clinical trials, patient demographics, and molecular profiling to suggest the most effective, individualized treatment regimens for patients^[53]^.

**2. AI in Chemotherapy and Radiation Therapy Planning**

AI can assist oncologists in planning chemotherapy and radiation therapies by predicting the optimal dose and schedule that would be most effective for a given patient while minimizing side effects. Machine learning algorithms can analyze data from past cases to predict how patients with similar tumor types and genetic profiles responded to specific treatments. This information can guide clinicians in adjusting treatment protocols for more effective and less toxic outcomes. AI is also useful in radiation therapy planning by helping to delineate tumor boundaries accurately in imaging scans, ensuring that radiation is delivered precisely to the cancerous tissue while sparing surrounding healthy tissue. AI-based tools can automatically segment and identify organs at risk, streamlining the planning process and reducing the possibility of errors^[54]^.

**3. AI in Treatment Monitoring**

AI-driven systems can be used for real-time monitoring of treatment response. By analyzing serial imaging studies or biomarkers, AI can help clinicians track tumor shrinkage or progression, potentially adjusting treatment regimens based on the observed effectiveness. This approach enables more dynamic and adaptive treatment strategies, ensuring that patients receive the most appropriate care throughout their treatment journey.

### Current limitations of AI-driven approaches in breast cancer diagnosis and treatment planning

While AI is making significant strides in breast cancer diagnosis and treatment, there are several challenges that hinder its full integration into clinical practice.

1. **Data Quality and Availability**

AI models rely heavily on large, high-quality datasets to train and make predictions. However, these datasets are not always representative of the diverse patient population, which can lead to biased outcomes. For instance, AI systems trained predominantly on data from certain ethnic groups may perform poorly when applied to patients from different backgrounds. Ensuring that datasets are diverse and inclusive is essential for improving the generalizability and accuracy of AI models.

**2. Regulatory and Ethical Concerns**

The integration of AI into clinical practice raises important regulatory and ethical issues. AI tools must undergo rigorous validation to ensure their safety and efficacy before being used in patient care. However, the regulatory processes for AI in healthcare are still evolving, and there is a lack of standardized guidelines for approval and implementation. Additionally, ethical concerns surrounding patient privacy, data security, and algorithmic transparency must be addressed to ensure that AI-driven approaches are used responsibly^[54]^.

**3. Interpretability and Trust in AI**

One of the key barriers to the widespread adoption of AI in breast cancer care is the lack of interpretability of some AI models. Many deep learning algorithms, for example, function as “black boxes,” meaning that they provide predictions without explaining how they arrived at those conclusions. This lack of transparency can be concerning for clinicians who need to understand the reasoning behind treatment recommendations, especially when making critical decisions. Building trust between clinicians and AI systems requires making these algorithms more interpretable and explainable^[55]^.

**4. Integration into Clinical Workflow**

Integrating AI into existing clinical workflows presents logistical challenges. Healthcare professionals need to be trained to use AI tools effectively, and the tools must be compatible with the existing electronic health records (EHR) systems. Furthermore, AI-generated recommendations must be viewed as complementary to, rather than replacing, the clinical judgment of healthcare providers. Ensuring that AI is seamlessly incorporated into daily practice without overwhelming clinicians is a critical hurdle^[56]^.

**5. Cost and Access**

While AI technologies hold promise, they can be expensive to develop, implement, and maintain. The costs associated with AI systems may limit their accessibility, particularly in low-resource settings. Furthermore, the availability of AI-driven diagnostic and treatment planning tools may be concentrated in high-income countries, leaving patients in other regions without access to these advancements.

### Liquid biopsies vs. traditional tissue biopsies in breast cancer detection and monitoring

The detection, diagnosis, and monitoring of breast cancer have traditionally relied on tissue biopsies, where a sample of tumor tissue is surgically or percutaneously collected and analyzed. However, with advances in molecular biology, liquid biopsies have emerged as a promising non-invasive alternative. Liquid biopsies involve analyzing biological fluids, primarily blood, to detect cancer-related biomarkers such as circulating tumor DNA (ctDNA), RNA, extracellular vesicles, or circulating tumor cells (CTCs). This innovative approach has shown great potential in detecting and monitoring breast cancer, offering a less invasive and more dynamic way to track the disease. However, there are key differences between liquid biopsies and traditional tissue biopsies, particularly in terms of accuracy, sensitivity, and clinical applicability. This narrative explores the advantages and limitations of both methods and discusses how liquid biopsies compare to traditional tissue biopsies in breast cancer detection and monitoring^[57]^.

**Traditional Tissue Biopsies**: Tissue biopsies have long been considered the gold standard for diagnosing breast cancer and determining its molecular characteristics. A tissue biopsy involves obtaining a sample from a suspicious mass, often guided by imaging techniques like ultrasound, mammography, or MRI. This tissue is then examined histologically and molecularly to confirm the presence of cancer, determine the subtype, assess hormone receptor status (e.g., estrogen receptor, progesterone receptor), and evaluate HER2 (human epidermal growth factor receptor 2) expression. The information gathered from a tissue biopsy is critical in making treatment decisions, such as whether the patient should undergo chemotherapy, targeted therapy, or hormonal therapy^[58]^.

### Accuracy and sensitivity of tissue biopsies

Traditional tissue biopsies are considered highly accurate for diagnosing breast cancer and assessing tumor characteristics at a specific point in time. They provide direct insights into the tumor microenvironment, which allows for detailed analysis of tumor grade, molecular markers, and the presence of genetic mutations. This precision is crucial for selecting personalized treatments and predicting patient prognosis. However, tissue biopsies have some limitations. The accuracy of the biopsy depends on factors such as the size, location, and accessibility of the tumor, and the procedure can sometimes be painful or risky, especially for tumors located in difficult-to-reach areas. Additionally, tissue biopsies provide a snapshot of the tumor at the time of sampling, but they may not reflect the full genetic or molecular heterogeneity of the tumor. Tumors can evolve over time, with subclones emerging that may not be present in the sampled tissue, potentially limiting the effectiveness of the treatment chosen based on that sample^[59]^.

### Liquid biopsies: a revolutionary alternative

Liquid biopsies, on the other hand, offer a non-invasive way to detect cancer-related biomarkers through the analysis of bodily fluids, most commonly blood. Liquid biopsies have gained traction due to their ability to detect key cancer markers, such as ctDNA, CTCs, and exosomes, all of which can be found in circulation due to the shedding of tumor cells or their byproducts into the bloodstream.

### Accuracy and sensitivity of liquid biopsies

One of the key advantages of liquid biopsies is their ability to detect cancer-related biomarkers across the entire tumor, providing a more comprehensive view of the tumor’s molecular landscape compared to a single tissue sample. ctDNA, for instance, can reveal genetic mutations, structural variations, and epigenetic changes across the entire tumor, offering insights into tumor heterogeneity that are often missed in tissue biopsies. Liquid biopsies also allow for monitoring of treatment response and detection of minimal residual disease (MRD) or recurrence, which is critical for adjusting therapeutic strategies. In terms of sensitivity, liquid biopsies have shown promise in early cancer detection, even in cases where the tumor is small or difficult to reach. ctDNA levels tend to rise as the tumor grows, allowing for earlier detection compared to tissue biopsies, which may only capture cancer at later stages when the tumor has reached a size large enough to be sampled. Moreover, liquid biopsies can detect circulating biomarkers that might be missed by traditional tissue biopsies, including cancer subclones that evolve over time. However, liquid biopsies are not without their challenges. The accuracy and sensitivity of liquid biopsies depend heavily on the specific biomarker being measured. ctDNA, for example, can be difficult to detect in early-stage cancers or in cases where the tumor is not shedding significant amounts of DNA into the bloodstream. Moreover, the sensitivity of liquid biopsies can vary depending on the assay used, and false negatives can occur, particularly in patients with low tumor burden or when the tumor’s genetic mutations are not well represented in ctDNA^[60]^.

### Clinical applicability: liquid biopsy vs. traditional biopsy

1. **Non-Invasiveness and Repeated Monitoring**

One of the most significant advantages of liquid biopsies over traditional tissue biopsies is their non-invasive nature. Liquid biopsies can be performed through a simple blood draw, making them far less invasive, painful, and risky for patients. This is particularly important for patients who may need frequent monitoring throughout their treatment journey. For example, liquid biopsies can be used to track tumor dynamics, detect early signs of recurrence, or assess treatment efficacy without the need for repeated invasive procedures. Traditional tissue biopsies, while invaluable in providing direct tissue samples, can be more challenging to perform repeatedly due to their invasive nature. For patients undergoing treatment for metastatic breast cancer, frequent tissue biopsies are often not feasible or recommended. Liquid biopsies, therefore, offer an attractive alternative for real-time monitoring, allowing clinicians to detect changes in tumor characteristics and adjust treatment plans accordingly^[61]^.

**2. Detection of Minimal Residual Disease and Recurrence**

Liquid biopsies have shown promise in detecting minimal residual disease (MRD) and cancer recurrence. MRD refers to the small number of cancer cells that may remain in a patient’s body after treatment and can later lead to relapse. While tissue biopsies may not be able to detect MRD due to the low number of residual cancer cells, liquid biopsies can identify even small amounts of ctDNA or CTCs circulating in the blood, offering a potential tool for early detection of recurrence. In contrast, tissue biopsies are typically less effective in detecting MRD since they require the presence of a detectable tumor mass. Liquid biopsies, therefore, have the advantage of offering more timely insights into disease progression and recurrence, which can significantly improve patient outcomes through earlier intervention^[60]^.

**3. Limitations of Liquid Biopsy in Clinical Practice**

Despite the many benefits, liquid biopsies still face several challenges that limit their widespread clinical use. First, the sensitivity of liquid biopsies can be lower than that of traditional tissue biopsies, especially in cases where the tumor is small, or the genetic mutations are not readily detectable in ctDNA or CTCs. Moreover, the clinical interpretation of liquid biopsy results remains complex, as the relationship between ctDNA levels and tumor burden is not always straightforward. Second, while liquid biopsies can provide valuable insights into the tumor’s genetic and molecular landscape, they are not yet as reliable as tissue biopsies for determining the exact tumor subtype, grading, or hormone receptor status. Certain markers, such as HER2 amplification or estrogen receptor expression, are typically assessed through tissue samples, and liquid biopsy technology may not be able to provide this level of detail^[61]^.

## Genetic insights shaping personalized treatment strategies in breast cancer

Breast cancer, one of the most common malignancies worldwide, is a heterogeneous disease, meaning that it varies widely in terms of genetic mutations, tumor biology, and response to treatment. Over the years, advances in genomics have revolutionized the understanding of breast cancer at the molecular level. Genetic insights into the tumor’s DNA, RNA, and protein expressions have laid the foundation for personalized treatment strategies, where therapy is tailored based on the unique genetic profile of a patient’s cancer. Personalized treatment, or precision medicine, allows for a more targeted, effective approach that can minimize side effects and improve outcomes. Central to this shift in treatment paradigms are key genetic discoveries that guide the selection of targeted therapies. This narrative explores how genetic insights have influenced breast cancer treatment strategies and the specific role they play in guiding targeted therapy selection^[62]^.

### The role of genetic insights in breast cancer

Breast cancer is not a single disease but a collection of subtypes with distinct molecular characteristics. These subtypes are primarily defined by the presence or absence of certain genetic mutations and biomarkers, which play a significant role in determining the aggressiveness of the disease and its response to treatment. Understanding the genetic makeup of breast cancer allows for better risk stratification and more precise targeting of therapies, minimizing unnecessary treatments and optimizing clinical outcomes. The major genetic insights that have influenced personalized treatment strategies in breast cancer include the identification of key oncogenes, tumor suppressor genes, and specific mutations. These insights are critical in selecting targeted therapies that block the molecular pathways driving tumor growth^[63]^.

1. **Hormone Receptor Status**

One of the most influential genetic insights in breast cancer is the identification of hormone receptor status, specifically the presence of estrogen receptors (ER) and progesterone receptors (PR) on the tumor cells. Tumors that are ER-positive (ER+) and PR-positive (PR+) tend to grow in response to hormones like estrogen and progesterone. These tumors represent about 70–80% of all breast cancers and are generally more responsive to hormonal therapies. Hormonal therapies, such as selective estrogen receptor modulators (SERMs) like tamoxifen or aromatase inhibitors, work by either blocking estrogen from binding to its receptor or lowering the levels of estrogen in the body. The identification of ER and PR expression in breast cancer is thus one of the most important genetic insights for selecting therapy. Patients with ER + breast cancer are typically treated with endocrine therapy, which has proven to significantly improve survival and reduce recurrence rates^[64]^.

On the other hand, tumors that are ER-negative (ER−) and PR-negative (PR–) are less likely to respond to hormonal therapies and often require different treatment strategies, including chemotherapy and targeted therapies.

**2. HER2/Neu Amplification**

Another landmark genetic discovery in breast cancer is the amplification of the HER2 gene, which encodes for a protein involved in cell growth and division. About 15–20% of breast cancers overexpress the HER2 protein, a condition known as HER2-positive breast cancer. HER2-positive tumors tend to be more aggressive, growing and spreading more quickly than HER2-negative tumors. The identification of HER2 amplification has led to the development of targeted therapies that specifically inhibit the HER2 pathway. The most well-known targeted therapies for HER2-positive breast cancer include monoclonal antibodies like trastuzumab (Herceptin) and small molecule inhibitors such as lapatinib. These therapies work by binding to the HER2 protein or blocking its signaling pathway, ultimately inhibiting tumor cell growth and promoting apoptosis (cell death). The advent of HER2-targeted therapies has dramatically improved the prognosis of patients with HER2-positive breast cancer, transforming a once poor prognosis into a more treatable condition. Genetic testing for HER2 amplification is now a standard part of breast cancer diagnosis and treatment planning^[65]^.

**3. BRCA1 and BRCA2 Mutations**

Mutations in the BRCA1 and BRCA2 genes are well-known genetic risk factors for breast cancer. These genes are involved in DNA repair, and when mutated, they increase the risk of developing not only breast cancer but also ovarian and other cancers. Women with inherited mutations in BRCA1 or BRCA2 have a significantly higher lifetime risk of developing breast cancer, often at younger ages. Genetic testing for BRCA mutations has profound implications for treatment decisions. For patients with BRCA1 or BRCA2 mutations, the development of PARP (poly ADP ribose polymerase) inhibitors like olaparib has provided a novel targeted treatment option. PARP inhibitors work by blocking DNA repair mechanisms in cancer cells, exploiting the fact that BRCA-mutated cells are already deficient in DNA repair. This strategy induces synthetic lethality, where the cancer cells cannot survive without proper DNA repair, leading to their death. In addition to PARP inhibitors, women with BRCA mutations may also benefit from other targeted therapies or preventive measures, such as prophylactic mastectomy or oophorectomy, to reduce cancer risk^[63]^.

**4. PI3K/Akt/mTOR Pathway Mutations**

The PI3K/Akt/mTOR signaling pathway is another critical pathway in breast cancer development. Mutations or amplifications in components of this pathway can lead to uncontrolled cell growth and resistance to chemotherapy. Genetic insights into the PI3K pathway have revealed that about 40% of breast cancer cases have alterations in these genes, particularly in estrogen receptor-positive (ER+) breast cancers. The identification of mutations in the PI3K pathway has led to the development of targeted therapies that inhibit these specific signaling pathways. One such drug is alpelisib, a PI3K inhibitor, which has shown promise in treating breast cancers with PIK3CA mutations, a common genetic alteration in ER+ breast cancers. Targeting the PI3K pathway in combination with other therapies, such as hormonal therapy, is emerging as a powerful strategy for overcoming resistance to endocrine therapy and improving outcomes in patients with advanced or metastatic ER+ breast cancer^[64]^.

**5. Gene Expression Profiling and Oncotype DX**

Gene expression profiling, using assays like Oncotype DX, has significantly advanced personalized treatment in breast cancer. Oncotype DX analyzes the expression of a set of genes in the tumor to predict the likelihood of recurrence and to guide the decision to administer chemotherapy. This test is particularly useful for patients with early-stage, ER-positive breast cancer, as it helps determine whether chemotherapy is necessary in addition to hormonal therapy. By assessing the risk of recurrence at a molecular level, Oncotype DX allows for more individualized treatment plans, sparing patients from unnecessary chemotherapy if their cancer is unlikely to recur^[65]^.

### The impact of technological innovations on early detection of breast cancer and patient outcomes

Breast cancer is one of the most common cancers diagnosed in women worldwide, and early detection remains one of the most critical factors influencing patient outcomes. With advancements in technology, the landscape of breast cancer diagnosis has undergone a profound transformation. Innovations in imaging techniques, genetic testing, artificial intelligence (AI), and liquid biopsies have made it possible to detect breast cancer at increasingly earlier stages, which is crucial for improving prognosis and treatment options. This narrative delves into how the integration of these technological innovations has contributed to earlier detection and how it has positively impacted patient outcomes^[66]^.

### Advancements in imaging technologies

One of the most significant technological innovations in breast cancer detection has been the development and refinement of imaging technologies. These include mammography, ultrasound, magnetic resonance imaging (MRI), and digital breast tomosynthesis (DBT), commonly known as 3D mammography.

1. **Mammography and Digital Mammography**

Mammography has long been the gold standard for breast cancer screening. Traditional film mammography was effective but had its limitations, particularly in dense breast tissue where it was difficult to detect tumors. With the introduction of digital mammography, images could be manipulated to enhance areas of concern, offering higher sensitivity and more accurate detection, particularly in women with denser breasts. The transition to digital mammography has significantly improved breast cancer detection, allowing for the identification of tumors at smaller sizes and earlier stages. It also enables better visualization of microcalcifications, which can be an early sign of breast cancer. Regular mammography screening has been shown to reduce breast cancer mortality rates by enabling the detection of cancer before it spreads to lymph nodes or other organs^[67]^.

**2. Digital Breast Tomosynthesis (DBT)**

DBT, or 3D mammography, is a newer technology that takes multiple X-ray images of the breast from different angles and reconstructs them into a three-dimensional image. This technology has been shown to reduce the problem of overlapping tissue, which can obscure tumors in traditional 2D mammography. DBT has been shown to increase the detection of invasive breast cancers and reduce false-positive results. Studies indicate that DBT can detect more cancers at an earlier stage, and its ability to identify cancers that might have been missed by traditional mammography leads to more effective and earlier interventions. Furthermore, DBT has improved the detection of smaller cancers and reduced recall rates, leading to fewer unnecessary biopsies and associated anxiety^[68]^.

**3. Magnetic Resonance Imaging (MRI)**

MRI is often used in combination with mammography, particularly for women at high risk of breast cancer due to genetic factors such as BRCA mutations. MRI uses powerful magnets and radio waves to create detailed images of the breast tissue, which is particularly useful for detecting tumors in dense breast tissue, where mammograms may not be as effective. MRI has been proven to be more sensitive than mammography in detecting early-stage breast cancers, particularly in women with dense breasts or those at high genetic risk. It can identify smaller tumors, sometimes even before they are detectable by other imaging modalities, thus allowing for earlier and more accurate diagnosis. For high-risk patients, MRI is increasingly becoming an essential tool in the surveillance and early detection of breast cancer^[69]^.

### Genetic Testing and Risk Stratification

Alongside advances in imaging, genetic testing has become a cornerstone of early breast cancer detection, especially for women with a family history or genetic predispositions to the disease. Testing for mutations in the BRCA1, BRCA2, and other cancer-related genes can provide crucial information about a woman’s likelihood of developing breast cancer, and it can also guide screening and prevention strategies.

1. **Genetic Testing and Early Screening**

Women who test positive for BRCA mutations have a significantly higher lifetime risk of developing breast cancer. As a result, genetic testing allows for more personalized screening plans, such as earlier or more frequent mammograms and MRIs. Genetic testing has contributed to early detection by enabling high-risk individuals to begin screening at younger ages. For example, women with BRCA mutations may start annual mammograms and MRIs in their 20s, which significantly increases the likelihood of detecting cancer at an earlier stage. Moreover, genetic testing has led to the use of preventive measures, such as prophylactic mastectomy or oophorectomy, in high-risk patients, further reducing cancer incidence and mortality^[66]^.

## Artificial intelligence and machine learning

Artificial intelligence (AI) has begun to play an increasingly pivotal role in breast cancer detection. AI systems, particularly those that leverage machine learning, are being trained to analyze mammograms, MRIs, and ultrasound images with high levels of precision.

1. **AI-Driven Imaging Analysis**

AI can analyze mammograms and other imaging results more quickly and, in some cases, more accurately than human radiologists. By using deep learning algorithms, AI can be trained to detect subtle patterns in breast tissue that may indicate the presence of cancer. This technology also assists in reducing human error, fatigue, and variability in readings. AI has demonstrated a significant ability to detect early-stage cancers, sometimes even before they are evident to human clinicians. AI systems have been shown to outperform radiologists in certain studies, particularly in detecting small or subtle tumors that might be missed during manual screening. With the assistance of AI, the process of diagnosing breast cancer becomes faster and more accurate, leading to earlier detection and improved patient outcomes^[65]^.

**2. Risk Prediction Models**

In addition to imaging analysis, AI is being used to develop sophisticated risk prediction models. These models combine imaging data with clinical information and genetic profiles to predict a patient’s likelihood of developing breast cancer. By assessing large datasets, AI algorithms can identify risk factors that may not be obvious to clinicians. AI-driven risk prediction models enable more personalized screening schedules. Women identified as high risk based on AI-generated assessments can be monitored more closely, and cancers can be detected at the earliest possible stages. This proactive approach has the potential to save lives by identifying tumors before they grow or metastasize^[67]^.

### Liquid biopsy: a non-invasive approach

Liquid biopsy is an emerging technology that analyzes tumor DNA or other markers circulating in a patient’s blood. Liquid biopsy is a non-invasive alternative to traditional tissue biopsy and holds promise for monitoring breast cancer progression and recurrence.

1. **Detection of Early-Stage Cancers**

In some cases, liquid biopsies have shown promise in detecting breast cancer even in its early stages, offering an alternative to imaging and tissue biopsy. By detecting specific mutations or genetic alterations present in tumor DNA, liquid biopsy can identify cancer in its earliest form before the appearance of clinical symptoms. Liquid biopsies offer a non-invasive, quick, and repeatable method for early cancer detection. As this technology evolves, it holds the potential to become a routine screening tool, particularly for patients at high risk for breast cancer. Liquid biopsy could complement traditional imaging techniques, allowing for earlier detection and ongoing monitoring of patients with a history of breast cancer or those at high risk^[68]^.

### Impact on patient outcomes

The integration of technological innovations into breast cancer detection has had a profound impact on patient outcomes. Early detection, made possible through advanced imaging techniques, genetic testing, AI-driven analysis, and liquid biopsy, allows for earlier intervention and more personalized treatment strategies. This, in turn, leads to:

1. **Improved Survival Rates**: Early detection of breast cancer, particularly when the cancer is confined to the breast or nearby lymph nodes, leads to a higher chance of successful treatment. Patients diagnosed at earlier stages often experience better long-term survival rates compared to those diagnosed at more advanced stages.
2. **Reduced Treatment Intensity and Side Effects**: Early-stage cancers are often treated with less aggressive therapies, reducing the risk of side effects. This not only improves quality of life but also reduces healthcare costs associated with more intensive treatments for advanced cancers.
3. **Increased Access to Targeted Therapies**: With earlier detection, patients can benefit from targeted therapies that are more effective at treating specific genetic mutations or molecular characteristics of their cancer. This results in better treatment responses and fewer adverse effects^[69]^.

## Advancements in breast cancer research: translating into improved long-term survival and quality of life for patients

Over the past few decades, significant advancements in breast cancer research have dramatically changed the landscape of treatment and patient outcomes. Thanks to a combination of improved detection methods, better understanding of cancer biology, and more targeted therapies, survival rates for breast cancer patients have steadily increased. Beyond merely extending life, research has also focused on enhancing the quality of life for survivors, ensuring they not only live longer but also thrive after treatment. This narrative explores how these advancements in breast cancer research translate into better long-term survival and quality of life for patients^[28]^.

1. **Early Detection and Improved Screening Techniques**

The cornerstone of improved breast cancer outcomes is early detection, which allows for more effective and less invasive treatments. Research into better screening tools, such as advanced mammography, 3D breast imaging (tomosynthesis), and magnetic resonance imaging (MRI), has led to earlier identification of tumors that are smaller, less aggressive, and confined to the breast tissue. Liquid biopsies, which detect genetic mutations and tumor DNA circulating in the blood, are also emerging as a non-invasive tool for early detection, particularly in high-risk patients or those with metastatic disease^[29]^.

### Impact on survival

Early detection leads to a higher likelihood of identifying cancer before it has spread to lymph nodes or distant organs, which is crucial for initiating treatments that can result in a cure or long-term remission. As a result, survival rates have increased significantly for breast cancer patients diagnosed at early stages.

### Impact on quality of life

Earlier diagnosis typically results in less aggressive treatments, sparing patients from the harsh side effects of chemotherapy and reducing the need for extensive surgery. This contributes to an improved quality of life during and after treatment.

**2. Personalized and Targeted Therapies**

Another major leap in breast cancer treatment has been the shift from generalized chemotherapy to personalized and targeted therapies. Researchers have made significant strides in understanding the molecular and genetic underpinnings of different breast cancer subtypes, such as HER2-positive, triple-negative, and hormone receptor-positive cancers. This has paved the way for treatments that specifically target the cancer’s genetic mutations or hormonal pathways, rather than using broad-spectrum chemotherapy, which indiscriminately kills both cancerous and healthy cells^[30]^.

- **HER2-targeted therapies** like trastuzumab (Herceptin) have revolutionized the treatment of HER2-positive breast cancer, improving survival rates and reducing the risk of recurrence.
- **Hormone therapies** such as tamoxifen and aromatase inhibitors have drastically reduced recurrence rates in hormone receptor-positive breast cancers, which are sensitive to estrogen.
- **Immunotherapy and PARP inhibitors** are now showing promise for patients with triple-negative breast cancer, a subtype historically known for its poor prognosis^[31]^.

### Impact on survival

Personalized treatments tailored to the specific genetic profile of the tumor have led to more effective management of the disease, especially in cases where traditional therapies had limited success. Targeted therapies have significantly reduced recurrence rates, leading to improved long-term survival for many patients.

### Impact on quality of life

Targeted therapies tend to be less toxic than traditional chemotherapy, which often comes with debilitating side effects such as hair loss, nausea, and immunosuppression. As a result, patients experience fewer disruptions in their daily lives and can continue to work, engage in social activities, and care for their families. Additionally, the precision of targeted therapies means that they often focus on eradicating cancer cells while sparing healthy tissues, leading to fewer long-term side effects such as chronic fatigue, neuropathy, and organ damage.

**3. Advances in Surgical Techniques**

Surgical techniques for breast cancer treatment have also evolved, with the goal of removing tumors while preserving as much of the breast tissue and cosmetic appearance as possible. Research into breast-conserving surgeries, such as lumpectomy, combined with radiation therapy, has reduced the need for mastectomies in many cases^[32,33]^.

### Impact on survival

Breast-conserving surgery followed by radiation therapy has been shown to offer survival rates comparable to those of mastectomy for many patients. The ability to remove cancer while preserving the breast has led to a more favorable long-term outcome, both in terms of survival and psychological well-being.

### Impact on quality of life

Patients who undergo breast-conserving surgery often experience a better body image and less psychological distress compared to those who have mastectomies. This, in turn, leads to improved emotional well-being and a greater sense of self-esteem and confidence, which are important components of quality of life during survivorship.

**4. Supportive Care and Survivorship Programs**

Research has increasingly recognized that cancer treatment extends beyond the physical elimination of the disease. Long-term survivorship has become a focal point, with research aimed at improving the overall well-being of patients after the completion of cancer treatment. This includes psychological support, rehabilitation, and addressing the long-term side effects of treatment, such as fatigue, lymphedema, and cognitive impairments (often referred to as “chemo brain”).

- **Psychosocial support**: Programs that provide mental health services, counseling, and peer support groups have been shown to reduce depression, anxiety, and post-traumatic stress disorder (PTSD) among breast cancer survivors.
- **Exercise and rehabilitation**: Studies have shown that exercise interventions can reduce fatigue, improve cardiovascular health, and increase mobility for breast cancer survivors. These programs have been linked to better physical functioning and emotional well-being.
- **Survivorship care plans**: Research into survivorship care has led to comprehensive care plans that help patients transition from active treatment to post-treatment care, addressing health maintenance, psychological support, and monitoring for recurrence^[34,35]^.

### Impact on survival

Survivorship programs that focus on the early detection of recurrence and secondary cancers contribute to longer-term survival by ensuring that any signs of cancer are detected promptly and managed effectively.

### Impact on quality of life

The holistic approach to care, addressing not just the cancer but the physical, emotional, and social needs of patients, significantly improves quality of life. By providing patients with the tools to manage side effects, stay physically active, and access emotional support, these programs reduce the long-term burden of cancer treatment on mental health and physical functioning.

**5. Innovations in Radiotherapy and Minimally Invasive Techniques**

Research into advanced radiotherapy techniques, such as intensity-modulated radiation therapy (IMRT) and proton therapy, has also contributed to more precise treatment options. These technologies allow radiation to be delivered with greater accuracy, minimizing damage to surrounding healthy tissue and reducing side effects^[36]^.

### Impact on survival

Improved radiotherapy techniques can reduce the risk of recurrence by effectively targeting cancer cells while minimizing damage to normal tissues, leading to better long-term survival outcomes.

### Impact on quality of life

By sparing healthy tissues and reducing side effects like skin irritation, fatigue, and gastrointestinal distress, these advancements lead to a less disruptive treatment experience for patients. Furthermore, minimizing side effects such as lymphedema and heart or lung damage (especially important for women with left-sided breast cancer) improves both physical health and quality of life.

**6. Advancements in Clinical Trials and Research Infrastructure**

The growing emphasis on clinical trials and translational research has allowed for the rapid development and testing of new drugs, therapies, and treatment regimens. The integration of patient-reported outcomes into clinical trials has also allowed researchers to more effectively capture the impact of treatments on patients’ quality of life, ensuring that new therapies do not just extend survival but also improve daily functioning and well-being^[37,38]^.

### Impact on survival:

Clinical trials have been instrumental in identifying new treatment options that significantly improve survival rates, especially for patients with advanced or metastatic breast cancer. Additionally, new targeted therapies and immunotherapies emerging from clinical trials offer hope for patients whose cancer previously had limited treatment options.

### Impact on quality of life

By focusing on both efficacy and quality of life in clinical trials, researchers are developing treatments that allow patients to live longer without compromising their daily well-being. This balanced approach results in not only longer survival but also a more fulfilling life post-treatment.

### Implications of hypoxia in breast cancer

Hypoxia significantly influences the progression, aggressiveness, and treatment resistance of breast cancer. The oxygen-deprived tumor microenvironment orchestrates a series of adaptive changes in tumor biology, leading to profound clinical and pathological consequences.

1. **Tumor Progression and Aggressiveness**

Hypoxia promotes tumor growth and progression by facilitating angiogenesis, metabolic reprogramming, and immune evasion. The stabilization of HIFs drives the expression of pro-survival and pro-metastatic genes. For instance, increased levels of VEGF stimulate the formation of new, albeit aberrant, blood vessels to sustain tumor growth. However, these vessels are often poorly functional, perpetuating the hypoxic state and creating a vicious cycle that supports continuous tumor expansion. Additionally, hypoxia-induced EMT enhances cellular motility and invasiveness, increasing the likelihood of metastasis^[25]^.

**2. Therapy Resistance**

Hypoxia contributes to resistance against conventional breast cancer therapies, including chemotherapy, radiotherapy, and targeted treatments. Radiotherapy relies on oxygen to generate reactive oxygen species (ROS) that induce DNA damage in cancer cells. In hypoxic conditions, the reduced oxygen availability diminishes ROS production, making radiotherapy less effective. Chemotherapy is similarly impaired as hypoxia-induced dormancy in cancer cells reduces the efficacy of drugs targeting actively dividing cells. Hypoxia-driven upregulation of drug efflux pumps, such as P-glycoprotein, further limits chemotherapeutic success. Moreover, hypoxia-induced stemness and the expansion of cancer stem cell populations render tumors more resilient to treatments, contributing to recurrence^[26]^.

**3. Metastatic Potential**

The hypoxic tumor microenvironment is a key driver of breast cancer metastasis. Hypoxia-induced EMT downregulates epithelial markers, such as E-cadherin, and upregulates mesenchymal markers, including vimentin and N-cadherin. These changes allow tumor cells to detach from the primary tumor and invade surrounding tissues. Additionally, hypoxia modulates the extracellular matrix (ECM) by increasing the expression of matrix metalloproteinases (MMPs), which degrade ECM components and facilitate invasion. The acidic and immunosuppressive microenvironment created by hypoxia further promotes metastatic dissemination to distant organs, such as the lungs, liver, and brain^[27]^.

**4. Altered Immune Response**

Hypoxia has profound effects on the immune landscape of breast cancer. It suppresses anti-tumor immunity by impairing the activity of cytotoxic T cells and NK cells while promoting the recruitment of immunosuppressive cells, such as regulatory T cells (Tregs) and MDSCs. Hypoxia-induced expression of immune checkpoint molecules, such as programmed death-ligand 1 (PD-L1), dampens immune surveillance, allowing cancer cells to evade detection and destruction. These changes create an immune-privileged environment that favors tumor survival and growth^[28]^.

**5. Prognostic Significance**

The presence of hypoxia in breast cancer is associated with poor clinical outcomes. High levels of HIF-1α and other hypoxia markers correlate with increased tumor grade, advanced stage, and reduced overall survival. Hypoxia not only predicts resistance to therapy but also serves as an indicator of metastatic potential and disease recurrence. Its prognostic significance underscores the importance of integrating hypoxia assessment into clinical practice to better stratify patients and personalize treatment strategies^[29]^.

**6. Epigenetic Modifications and Tumor Heterogeneity**

Hypoxia induces epigenetic changes that contribute to tumor heterogeneity and adaptability. Hypoxia-induced microRNAs (e.g., miR-210) regulate key processes such as angiogenesis, metabolism, and apoptosis. DNA methylation and histone modifications mediated by hypoxia also alter gene expression patterns, leading to the selection of aggressive and therapy-resistant clones. This genomic and epigenetic plasticity complicates treatment and enhances tumor adaptability to changing environmental conditions^[30]^.

**7. Impact on Patient Quality of Life**

Hypoxia-driven breast cancer progression and treatment resistance adversely affect patients’ quality of life. Tumor hypoxia contributes to complications such as pain, fatigue, and cachexia, which are common in advanced disease stages. The aggressive behavior of hypoxic tumors often necessitates more intensive treatments, leading to increased side effects and psychological stress for patients^[31]^.

**8. Therapeutic Opportunities**

The implications of hypoxia have spurred interest in developing therapies targeting hypoxia and its downstream pathways. Strategies such as HIF inhibitors, VEGF blockers, and agents targeting metabolic adaptations (e.g., glycolysis inhibitors) hold promise for mitigating hypoxia-driven tumor progression. Combining these approaches with existing therapies may enhance treatment efficacy and improve outcomes for breast cancer patients^[32]^.

**9. Role in Personalized Medicine**

**Figure 3.**
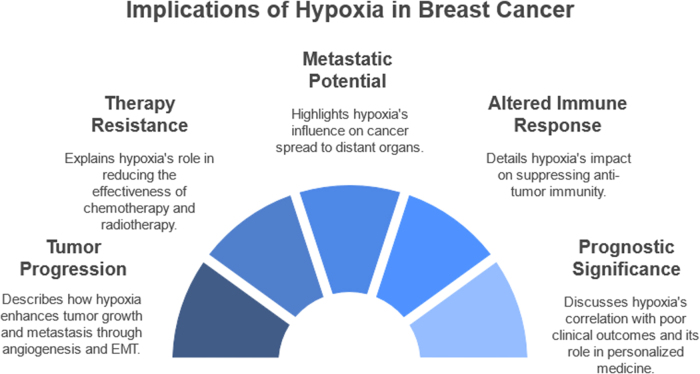
Implications of hypoxia in breast cancer.

Hypoxia biomarkers, such as HIF-1α, VEGF, and lactate levels, are emerging as tools for personalized medicine in breast cancer. Monitoring these biomarkers can help identify patients with hypoxic tumors and guide the selection of targeted therapies. Hypoxia imaging techniques, including PET scans using hypoxia tracers, further facilitate real-time assessment of tumor oxygenation, aiding in treatment planning and monitoring^[33,34]^ (Figure 3).

## Therapeutic strategies targeting hypoxia in breast cancer

Targeting hypoxia in breast cancer represents a promising approach to mitigate its detrimental effects on tumor progression, therapy resistance, and metastasis. Various strategies aim to modulate hypoxia directly or indirectly by targeting the signaling pathways, metabolic adaptations, and microenvironmental changes it induces. These therapeutic interventions are critical for improving treatment efficacy and patient outcomes.

1. **Hypoxia-Inducible Factor (HIF) Inhibitors**

HIFs are central mediators of hypoxia responses, making them prime therapeutic targets. Small molecules, such as PX-478, inhibit HIF-1α expression or activity, thereby suppressing downstream hypoxia-induced processes like angiogenesis and metabolic reprogramming. Acriflavine, a HIF dimerization inhibitor, prevents the interaction between HIF-α and HIF-β, blocking transcriptional activation of hypoxia-responsive genes. Clinical trials are evaluating the efficacy of these inhibitors in combination with conventional therapies to overcome hypoxia-driven resistance^[35]^.

**2. Anti-Angiogenic Agents**

Given the critical role of hypoxia-induced VEGF in angiogenesis, anti-angiogenic agents have been widely explored. Bevacizumab, a monoclonal antibody against VEGF, disrupts angiogenesis and normalizes tumor vasculature, improving oxygen delivery and therapeutic response. However, its clinical efficacy in breast cancer has been mixed, partly due to the development of resistance and compensatory mechanisms. Combining anti-angiogenic therapies with HIF inhibitors or immunotherapies may enhance their effectiveness^[36]^.

**3. Hypoxia-Activated Prodrugs (HAPs)**

HAPs are designed to exploit the hypoxic tumor microenvironment by becoming activated under low oxygen conditions. Agents such as tirapazamine and evofosfamide generate cytotoxic metabolites in hypoxic regions, selectively targeting hypoxic tumor cells while sparing normal tissues. These prodrugs show potential when combined with radiotherapy or chemotherapy, addressing the therapy-resistant hypoxic tumor core^[37]^.

**4. Metabolic Modulators**

Hypoxia-induced metabolic reprogramming, particularly the reliance on glycolysis, provides another avenue for therapeutic intervention. Glycolysis inhibitors, such as 2-deoxy-D-glucose (2-DG) and lonidamine, reduce glucose uptake and ATP production, selectively targeting hypoxic cancer cells. Similarly, inhibitors of lactate dehydrogenase A (LDHA) and monocarboxylate transporters (MCTs) disrupt lactate metabolism, reducing acidification of the tumor microenvironment and impairing tumor survival^[38]^.

**5. Oxygenation Strategies**

Improving oxygen delivery to hypoxic tumors is a direct approach to counteract hypoxia. Hyperbaric oxygen therapy (HBOT) increases oxygen availability in the bloodstream, enhancing radiotherapy efficacy by increasing reactive oxygen species (ROS) production. Additionally, erythropoietin-stimulating agents and blood transfusions can improve tumor oxygenation in specific clinical scenarios. However, these strategies require careful monitoring to avoid promoting angiogenesis or tumor growth^[39]^.

**6. Epigenetic Modulators**

Hypoxia induces epigenetic modifications that regulate tumor gene expression and behavior. Agents targeting DNA methyltransferases (e.g., azacitidine) or histone deacetylases (e.g., vorinostat) can reverse these modifications, restoring normal gene expression and sensitizing tumors to therapy. Hypoxia-induced microRNAs (e.g., miR-210) are also potential targets, with antagomirs and miRNA mimics being explored for therapeutic applications^[40]^.

**7. Immunotherapy Combinations**

The immunosuppressive microenvironment created by hypoxia impairs immune responses against tumors. Combining hypoxia-targeting strategies with immune checkpoint inhibitors, such as anti-PD-1/PD-L1 therapies, enhances anti-tumor immunity. Additionally, reducing hypoxia may improve immune cell infiltration and activation, making the tumor more susceptible to immunotherapy^[41]^.

**8. Nanoparticle-Based Therapies**

Nanoparticles offer a versatile platform for delivering hypoxia-targeted therapies. Oxygen-carrying nanoparticles, such as those encapsulating perfluorocarbon, can reoxygenate tumors, enhancing the efficacy of radiotherapy. Hypoxia-responsive nanoparticles can also release therapeutic agents specifically in hypoxic regions, minimizing off-target effects and improving drug efficacy^[42]^.

**9. Radiation Sensitizers**

Radiation sensitizers enhance the effectiveness of radiotherapy in hypoxic tumors. Agents like nimorazole improve oxygen utilization, increasing the sensitivity of cancer cells to radiation-induced DNA damage. These sensitizers are particularly beneficial in hypoxic breast tumors that are otherwise resistant to standard radiotherapy^[43]^.

**10. Combined Modality Approaches**

**Figure 4.**
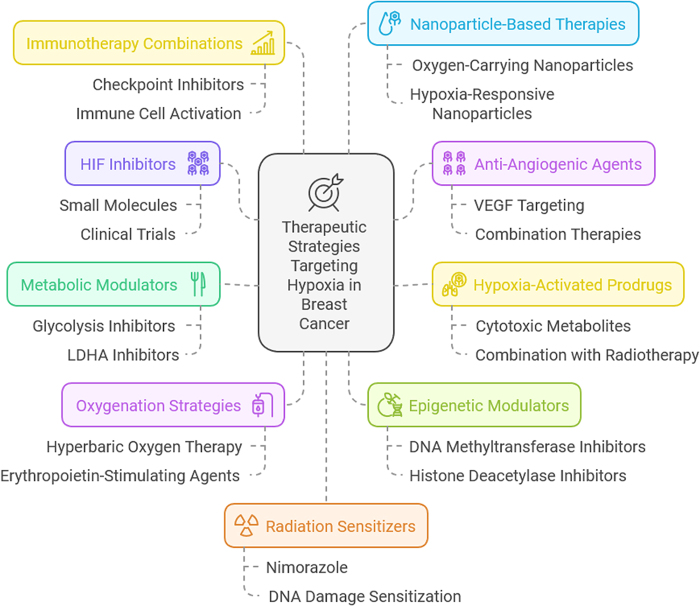
Therapeutic strategies targeting hypoxia in breast cancer.

Integrating hypoxia-targeting strategies with existing therapies has shown promise in preclinical and clinical settings. For instance, combining HIF inhibitors with chemotherapy addresses drug resistance, while pairing metabolic modulators with immunotherapy improves immune cell function. Multi-targeted approaches may also address the heterogeneity and adaptability of hypoxic tumors, providing more durable therapeutic responses^[44]^ (Figure 4).

## The ethical and economic implications of widespread adoption of precision medicine and advanced technologies in breast cancer treatment

The advent of precision medicine and advanced technologies in breast cancer treatment has ushered in a new era of personalized care, offering patients treatments that are tailored to their unique genetic makeup, tumor biology, and individual needs. These innovations hold the promise of more effective and less toxic therapies, improved survival rates, and enhanced quality of life for patients. However, as with any transformative shift in healthcare, the widespread adoption of these advancements brings with it a host of ethical and economic considerations.^[57]^

1. **Ethical Implications**

While the benefits of precision medicine are undeniable, the widespread implementation of such technologies presents several ethical concerns, particularly around issues of access, equity, informed consent, and data privacy.

### Equity and access to care

One of the most pressing ethical concerns regarding precision medicine is the potential for inequitable access to these advanced treatments. Precision therapies often require access to sophisticated diagnostic tools, genetic testing, and targeted drugs, which may not be available in all healthcare settings, particularly in low-resource or rural areas. This disparity in access could exacerbate existing health inequities, with wealthier patients and those in developed countries having greater access to cutting-edge treatments, while others remain reliant on more traditional, less effective options^[58,59]^.

### Informed consent and genetic privacy

With the integration of genetic testing and the use of genetic data in treatment decision-making, issues surrounding informed consent and privacy become crucial. Patients must be fully informed of the implications of undergoing genetic tests, not only in terms of their treatment options but also the potential for uncovering genetic predispositions to other diseases or conditions. Furthermore, the question of how genetic data is stored, shared, and used by healthcare providers and research institutions raises concerns about data privacy and the potential for misuse^[68]^.

### Personalization vs. standardization of care

As precision medicine tailors treatments to individual patients, it also challenges the traditional “one-size-fits-all” approach to medicine. While personalized care promises better outcomes for many, it also raises the question of whether this shift could undermine the principles of equity and fairness in healthcare. Standardized treatment protocols, which are designed to ensure consistency and fairness, may be seen as less relevant in the era of personalized medicine. This shift may create disparities between patients who have access to individualized therapies and those who do not, further complicating the ethical landscape.

**2. Economic Implications**

While precision medicine offers significant potential for improving patient outcomes, the economic implications of its widespread adoption are considerable. The cost of advanced technologies, genetic testing, and targeted therapies is a major concern, as these innovations are often more expensive than traditional treatment options. These increased costs could have far-reaching consequences for healthcare systems, insurance providers, and patients.

## Cost of treatment

Precision medicine often requires cutting-edge technologies, such as advanced genetic sequencing, biomarker testing, and personalized drug therapies, which can be prohibitively expensive. Targeted therapies and immunotherapies, while highly effective, come with high price tags that may not be affordable for all patients. The cost of these treatments may also place a significant financial burden on healthcare systems, especially in countries with universal healthcare, where governments bear the cost of providing care to large populations.

## Insurance coverage and reimbursement

The adoption of precision medicine raises complex questions about insurance coverage and reimbursement. Many insurance companies may be reluctant to cover expensive genetic testing or targeted therapies, particularly when the cost-benefit ratio of these treatments is still being evaluated. This could lead to disparities in access to care, with patients who can afford to pay out-of-pocket for these treatments receiving personalized care, while others are limited to more traditional, less effective therapies.

## Cost-effectiveness and healthcare resource allocation

The integration of precision medicine into breast cancer treatment may lead to the reallocation of healthcare resources, as more funds are directed toward high-cost treatments and technologies. While the potential for improved outcomes may justify the investment, questions arise about whether this allocation of resources could result in reduced funding for other critical areas of healthcare, such as prevention, public health initiatives, or treatment for other diseases.

## Long-term economic impact

While the upfront costs of precision medicine and advanced technologies are high, there is potential for long-term savings if these treatments lead to better patient outcomes, fewer hospitalizations, and reduced need for more aggressive interventions. By targeting therapies to the individual patient’s genetic profile, precision medicine has the potential to reduce the incidence of adverse drug reactions, avoid ineffective treatments, and increase the chances of achieving remission or long-term survival. This could ultimately result in a reduction in healthcare costs over time.

**3. Balancing Ethical and Economic Considerations**

The widespread adoption of precision medicine in breast cancer treatment will require a careful balance between ethical considerations and economic realities. Policymakers, healthcare providers, and researchers will need to work together to address issues of access, equity, and affordability, while ensuring that advancements in treatment do not exacerbate existing healthcare disparities. This balance will require the development of innovative solutions, such as subsidies for genetic testing, expanded insurance coverage, and policies that ensure equitable access to personalized therapies.

## Future perspectives in breast cancer

Breast cancer remains a dynamic and multifaceted challenge in oncology, requiring continuous innovation to address its complexities. The integration of emerging technologies, improved understanding of tumor biology, and a focus on personalized medicine are shaping the future of breast cancer diagnosis, treatment, and prevention. These advancements hold the promise of enhancing patient outcomes and minimizing disease burden.

1. **Precision Medicine and Genomic Profiling**

The era of precision medicine is revolutionizing breast cancer management by tailoring treatments to individual patient profiles. Comprehensive genomic profiling enables the identification of genetic mutations, epigenetic modifications, and molecular signatures that drive tumor progression. Biomarkers such as PIK3CA mutations, BRCA1/2 status, and HER2 expression guide therapeutic decision-making, allowing the selection of targeted therapies. Advancements in next-generation sequencing (NGS) and single-cell analyses are expected to uncover new actionable targets and mechanisms of resistance, further personalizing care^[45]^.

**2. Immunotherapy Advancements**

Immunotherapy is poised to play a more significant role in breast cancer treatment, particularly for triple-negative breast cancer (TNBC), which has limited therapeutic options. Immune checkpoint inhibitors (e.g., anti-PD-1/PD-L1) have shown promise in combination with chemotherapy. Efforts are underway to enhance immune responses through adoptive cell therapies, such as chimeric antigen receptor (CAR) T-cells, and cancer vaccines targeting breast cancer-specific antigens^[46]^.

**3. Liquid Biopsies and Early Detection**

Liquid biopsy technologies are transforming the detection and monitoring of breast cancer. These minimally invasive tests analyze circulating tumor DNA (ctDNA), exosomes, and circulating tumor cells (CTCs) to provide real-time insights into tumor dynamics. Liquid biopsies enable early detection, assessment of minimal residual disease (MRD), and monitoring of treatment efficacy. As technology advances, liquid biopsies could become a standard tool for personalized surveillance and intervention in breast cancer care^[47]^.

**4. Targeting the Tumor Microenvironment**

The tumor microenvironment (TME), comprising stromal cells, immune cells, and extracellular matrix components, plays a critical role in breast cancer progression. Therapies targeting TME factors such as hypoxia, angiogenesis, and immune suppression are gaining traction. Combining these approaches with systemic therapies may improve treatment responses by modulating the tumor ecosystem. Additionally, research into the role of the microbiome in the TME may unveil novel therapeutic targets^[48]^.

**5. Artificial Intelligence and Digital Pathology**

Artificial intelligence (AI) is transforming breast cancer diagnosis and treatment planning. AI-powered algorithms in digital pathology analyze histopathological images with high accuracy, improving diagnostic precision and reducing human error. AI also enhances radiological assessments by detecting subtle patterns in mammograms and MRI scans. In treatment planning, AI aids in predicting therapy responses and identifying optimal treatment regimens based on patient-specific data^[49]^.

**6. Advances in Radiation Therapy**

Innovations in radiation therapy are improving its precision and reducing associated toxicities. Techniques such as intensity-modulated radiation therapy (IMRT), proton therapy, and stereotactic body radiation therapy (SBRT) allow for highly targeted delivery of radiation, sparing surrounding healthy tissues. Hypoxia-targeted radiation sensitizers and imaging technologies are enhancing the effectiveness of radiotherapy in resistant breast cancer cases^[50,51]^.

**7. Nanotechnology in Drug Delivery**

Nanotechnology is revolutionizing drug delivery in breast cancer by improving the solubility, stability, and bioavailability of therapeutic agents. Nanoparticles and liposomes enable targeted delivery of drugs to tumor cells, reducing systemic toxicity and enhancing efficacy. Nanocarriers can also co-deliver multiple agents, such as chemotherapy and immunotherapy, to synergistically combat the disease. Advances in nanomedicine are expected to expand the arsenal of targeted therapies for breast cancer^[52]^.

**8. Epigenetic Therapies**

Epigenetic modifications, such as DNA methylation and histone acetylation, play a crucial role in breast cancer development and therapy resistance. Epigenetic therapies, including inhibitors of DNA methyltransferases (DNMTs) and histone deacetylases (HDACs), are under investigation to reverse aberrant gene expression. Combining epigenetic modulators with existing therapies may improve sensitivity to treatments and prevent tumor recurrence^[53]^.

**9. Survivorship and Quality of Life**

As survival rates improve, the focus on long-term survivorship and quality of life is becoming more prominent. Addressing the physical, emotional, and psychological challenges faced by breast cancer survivors is critical. Research into minimizing treatment-related side effects, such as lymphedema and cardiotoxicity, is ongoing. Rehabilitation programs, mental health support, and integrative care approaches are being developed to enhance survivorship experiences^[54]^.

**10. Preventive Strategies and Public Health Initiatives**

Advances in understanding breast cancer risk factors, including genetic predisposition and lifestyle influences, are driving preventive strategies. Prophylactic interventions, such as mastectomy for BRCA mutation carriers, are being complemented by chemoprevention using agents like tamoxifen and raloxifene. Public health initiatives focusing on awareness, screening, and equitable access to care aim to reduce breast cancer incidence and disparities in outcomes, particularly in underserved populations^[54]^.

## Conclusion

The landscape of breast cancer research and treatment is evolving rapidly, with numerous advancements on the horizon that offer hope for improved outcomes. From precision medicine and immunotherapy to innovations in early detection, targeted therapies, and personalized care, the future of breast cancer management is increasingly tailored to the unique characteristics of each patient and their tumor. By integrating genetic insights, technological innovations like artificial intelligence and liquid biopsies, and new therapeutic strategies, we can expect to achieve more accurate diagnoses, better treatment responses, and enhanced patient survival rates.

## Data Availability

Not applicable as this a narrative review.

## Research registration unique identifying number (UIN)

Not applicable as this a narrative review.
